# Molecular Phylogenetic Relationships Based on Mitogenomes of Spider: Insights Into Evolution and Adaptation to Extreme Environments

**DOI:** 10.1002/ece3.70774

**Published:** 2025-01-07

**Authors:** Rongxiang Zhang, Niyan Xiang, Xiaoman Gao, Guiyu Zhang, Tian Lu, Tao Yuan

**Affiliations:** ^1^ School of Biological Science Guizhou Education University Guiyang China; ^2^ School of Ecology and Environment Tibet Uneiversity Lhasa China; ^3^ School of Resources and Environmental Science Hubei University Wuhan China; ^4^ State Key Laboratory of Hybrid Rice, Laboratory of Plant Systematics and Evolutionary Biology, College of Life Sciences Wuhan University Wuhan China; ^5^ School of Municipal and Environmental Engineering Shandong Jianzhu University Jinan China

**Keywords:** adaptation, convergence, mitogenome, phylogeny, spider

## Abstract

In this study, we performed a comparative analysis based on a total of 255 spider mitogenomes and four outgroups, of which the mitogenomes of 39 species were assembled de novo, to explore the phylogenetic relationships and the adaptive evolution of mitogenomes. Results showed that *Argyroneta aquatica* had the longest mitochondrial length and the most pronounced codon preference to be UUA, followed by CCU. Codon usage frequencies were similar between families and codon usage in the mitogenome of spiders was mainly influenced by natural selection pressures rather than G/C mutation bias. Our phylogenetic topology clearly explained the evolutionary relationships among the spiders, and divergence time estimates indicated that the spiders originated in the early Devonian, and that the two clades of Mesothelae and Opisthothelae separated in the late Carboniferous. Ancestral range and trait reconstruction results supported the ancestral origin of spiders to the Devonian Nearctic realm, with the trapdoor being the original trait. Selection analysis detected positive selection signals in the *ATP8* gene in *Desis jiaxiangi*. The *ND5* gene is a convergent evolutionary gene between *D. jiaxiangi* and *A. aquatica* . Positive selection signals in the *ATP8* gene and convergent selection sites in the *ND5* gene may facilitate metabolic adaptation to the aquatic environment in two aquatic spiders. In conclusion, our analysis contributes to a better understanding of the taxonomic status, species diversity, mitochondrial characteristics, and environmental adaptations of these spiders.

## Graphical abstract

We used the largest dataset of mitogenomes available to reconstruct phylogenetic relationships and ancestral ranges and traits in spiders.

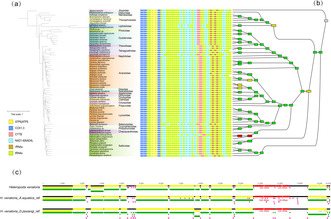

## 1 Introduction

Spiders (Araneae) and other arachnids constitute the subphylum Chelicerata under Arthropoda, belonging to a relatively large group of primitive animals. Spiders, which appeared approximately 400 mya (Magalhaes et al. 2020; Kallal et al. 2021), are extremely diverse, occupying terrestrial and some aquatic habitats on all continents, except Antarctica. The common features of spiders include the production of silk with associated spinners and venom glands. Up to seven different types of glands are present in extant spiders (Kovoor 1972, 1977), and some web‐building spiders have cribellar, which are short transverse fields of spigots and used to secrete a specialized type of silk (e.g., webs in the family Deinopidae). Spider silk is used for many tasks critical to spider biology and survival, such as constructing foraging webs (e.g., characterizing orb web), wrapping prey, building burrows, and producing egg sacs. As unique and extremely diverse carnivores, spiders are used as evolutionary models for studying key innovations and adaptive radiation hypotheses.

More than 51,000 species of spiders in 132 families and 4300 genera are known worldwide, but as of January 2024, the NCBI database (https://www.ncbi.nlm.nih.gov/nuccore/?term=mitochondrion%2C+complete+genome%2C+araneae) has reported only 89 mitogenomes and 31 nuclear genomes. Thus, the direction of spider phylogeny and evolution remain ambiguous. Many phylogenetic studies using morphological data to study spiders have been conducted over the past four decades, but many of the important nodes of the tree of life have not been resolved (Griswold et al. 2005; Fernandez et al. 2018). Multiple molecular marker phylogenies using nuclear genes in combination with mitochondrial genes constitute an early form of molecular phylogenetic research. Hausdorf (1999) published the first reconstructed molecular phylogenetic tree for spiders using a 900 bp 28S rRNA gene. Wheeler et al. (2017) constructed a phylogenetic tree of the order Arachnida, using six molecular markers from 932 spider samples from 115 families; they conducted one of the most comprehensive phylogenetic analyses in the order Arachnida to date. However, phylogenetic signals that can be represented by six molecular markers remain insufficient. Li et al. (2022) used 78 spider mitogenomes from 29 families to study spider phylogeny, gene rearrangements, and web evolution. Fernandez et al. (2018) used phylogenomics, comparative transcriptomics, and genealogical diversification analyses to study spider evolution, using transcriptome data from approximately 2500 genes from 159 species to construct spider phylogeny, divergence times, and ancestral state reconstruction of foraging webs. Gorneau et al. (2022) used four genes (*COI*, *16S*, *H3*, and *28S*) to construct a phylogenetic tree of 262 species of the family Sparassidae in 37 genera, which is the most comprehensive phylogenetic study of phylogeny and divergence time phylogeny of the family Sparassidae with data available. However, the phylogenetic framework of spiders is still subject to considerable uncertainty. With the diversification of molecular markers, the development of newer sequencing technologies, and the combination of traditional morphology with modern molecular phylogenetics and bioinformatics, numerous analytical methods have been integrated into the phylogeny, and the phylogenetic relationships of spiders have become clearer.

*Argyroneta aquatica* belongs to the family Dictynidae and the genus *Argyroneta*, which is the only one species in the world (Fan 2021), and is named after its life‐long life in the water. *A. aquatica* are mainly distributed in Inner Mongolia and Xinjiang in China, and abroad in the Mediterranean coastal countries in the Northern Hemisphere, Turkey and Iran in West Asia, and Japan and Korea in East Asia (Liu et al. 2015; Stefano, Riccardo, and Marco 2016; Fan 2021). *A. aquatica* usually live in streams, lakes and other environments with good water quality and abundant aquatic plants, and feed on small aquatic animals. As the only spider fully adapted to freshwater environments, it needs to overcome greater resistance and expend more energy swimming long distances to hunt for prey and find females for mating, and must overcome factors such as low temperatures and low oxygen (Fan 2021). *Desis jiaxiangi* belongs to the genus of *Desis* in the Desidae family, and it is mainly found in Australia, Brazil, China, the Galapagos Islands, India, Japan, New Zealand, Polynesia, and South Africa (Baehr, Raven, and Harms 2017; Li et al. 2021). Their ecological habitat, the intertidal zone, is one of the most stressful environments on earth, with dynamic changes in salinity, pH, temperature, and oxygen concentration (Li et al. 2021). It has been shown that *D. jiaxiangi* was capable of surviving for up to 19 days in anoxic underwater silk hides (Vink et al. 2017; Li et al. 2021). This unusual biology makes *D. jiaxiangi* amazing. Although water spiders and tide spiders are representatives of arachnid taxa that are adapted to extreme environments, the molecular mechanisms of their adaptation to extreme environments and the existence of convergent molecular mechanisms between the two species have been poorly investigated. Mitochondria serve as the yields of energy supply, and whether there are convergent molecular mechanisms of adaptation to extreme environments in low oxygen environments in the mitochondria of these two spider species has rarely been reported.

The mitogenomes of most metazoans usually consist of 15,000–17,000 bp‐long circular double‐stranded DNA molecules; each mitogenome contains 37 genes, a control region of variable length, 13 protein‐coding genes (PCGs), 22 tRNAs, and 2 rRNAs (Boore 1999). These PCGs are associated with oxidative phosphorylation, which is a key process in adenosine triphosphate (ATP) production, which in turn is essential for maintaining aerobic cellular respiration and energy supply. Mitochondrial genes evolve faster than nuclear genes in postnatal animals (Simon et al. 2006), and a high gene copy number ensures the stable inheritance of genetic material. Thus, mitogenomes have been widely used in phylogenetic reconstruction, population genetics, and evolutionary studies (Zhang et al. 2019; Wang et al. 2019). Given that mitogenomes have high resolution in phylogenetic studies, an increase in the amount of data will help to improve the resolution of phylogenetic relationships (Wang et al. 2018; Yu et al. 2019). Gene arrangement within arthropod mitogenomes is highly conserved. However, there are a number of invertebrate lineages in which the mitochondrial gene order is radically rearranged, as it is in insects (Cameron 2014). Previous studies have shown extensive gene rearrangements in spider mitoenomes, but a large portion of this rearrangement is due to annotation errors (Prada et al. 2023). Therefore, we reassembled 39 spider mitogenomes on the basis of NCBI database data and used published mitogenomes to reconstruct the phylogenetic history and analyze gene rearrangements of spiders. Using a total of 255 species from 66 families of Araneae, this study is the largest mitochondrial genomic analysis of spiders to date, aiming to resolve relationships between family‐level and some genus‐level orders of Araneae, and provide new data on the timing of the origin of numerous nodes. The problems of spider dispersal and web trait evolution were elucidated by reconstructing ancestral distribution areas and traits. We then conducted evolutionary analyses of specific taxa under the order Araneae to reveal the molecular basis of multiple adaptations in spiders. This study will lay the foundation for subsequent studies of the phylogenetic origins, dispersal, divergence histories, and environmental adaptations of some of the key spider populations.

## 2 Materials and Methods

### 2.1 Data Acquisition, Assembly, and Annotation

A total of 216 spider mitogenomes are currently indexed in the NCBI database (as of February 2024), and 87 species have complete annotation information. Therefore, we re‐annotated the remaining 129 unannotated mitogenomes along with the mitogenome of *Mastigoproctus giganteus* (outgroup). Then, we downloaded the SRA data of 39 spiders from the NCBI database (Appendix S1). First, we prioritized data generated by the Illumina sequencing platform to ensure the accuracy of the data. Second, we randomly extracted the data in SRA and compared them to the NCBI database data to determine whether the SRA data had species errors and other conditions. Low‐quality reads and the adaptors of the raw data were removed using Fastp with default settings (Chen et al. 2018). Mitogenome assembly was performed in MitoZ 2.3 (Meng et al. 2018), and annotation was performed in MitoFinder v1.4.1 (Allio et al. 2020) and Geneious Prime (Kearse et al. 2012). The gene identity was 65%. Finally, we manually checked all PCGs of the mitogenomes designed for this study to ensure that there were no annotation errors. Species information and accession numbers used in this study are listed in Appendix S1.

### 2.2 Comparative Genomic Analysis

Some spiders' genomes failed to loop and lacked good annotation. Therefore, only 76 well‐annotated and fully circular mitogenomes were selected for comparative analysis (Appendix S2). For codon preference analysis, we first used MAFFT software (Katoh and Standley 2013) to align PCGs, and then used FASconCAT‐G v1.04 (Kuck and Longo 2014) to link genes. The compositional skewness of each PCG in the mitogenome was calculated using the following formula: AT‐skew = (A − T)/(G + C), GC‐skew = (G − C)/(G + C). The relative synonymous codon usage (RSCU) was analyzed using CodonW v1.4.4 (Peden 2000), and GC content was analyzed using Geneious software (Kearse et al. 2012). An RSCU value of > 1.00 indicates that a codon is used more frequently than expected. The plots of the effective number of codons (ENC) are commonly used in assessing codon usage patterns in genes. The relationship between ENC and GC3s was visualized using R scripts (https://github.com/taotaoyuan/myscript). Predicted ENC values that lie on or above the expected curve can indicate that codon usage is primarily influenced by G + C mutations. However, if natural selection or other factors are considered, predicted ENC values will fall below the expected curve (Wright 1990). Average nucleotide identity (ANI) values of the 26 reference spider mitogenomes and two query mitogenomes ( *A. aquatica* and *D. jiaxiangi*) were determined through pairwise comparisons using fastANI with the parameter “‐‐minFrag 0.5 ‐k 16” (Richter et al. 2016). In addition, pairwise comparisons of mitogenomes were performed using the Common Interval Rearrangement Browser (CREx) (Bernt et al. 2007) to reconstruct gene order rearrangement events that may have occurred in spiders.

### 2.3 Phylogenetic Analysis and Estimation of Divergence Times

The complete or nearly complete mitogenomes (containing at least 12 PCGs) of 255 spiders from 66 families were used for phylogenetic analysis (Appendix S1), and four species (i.e., *Achelia bituberculata* , *Eremobates palpisetulosus* , *Pseudogarypus banksi* , and *M. giganteus* ) were used as outgroups. As in the results of Prada et al. (2023) and Moreno‐Carmona et al. (2021), tRNA genes had the highest number of errors in the annotation of the spider mitogenomes, followed by D‐loop structures. Therefore, we used PCGs in phylogenetic analyses. A total of 12 PCGs were extracted using PhyloSuite v1.2.1 (Zhang, Jiang, et al. 2020; Zhang, Gao, et al. 2020; *ATP8* was deleted because it was not found in some species) and then aligned using MAFFT L‐INS‐i (Katoh and Standley 2013) as described previously (Yu et al. 2023). The poorly aligned sequences were removed using the “automated1” parameter in TrimAl v1.4.1 (Capella‐Gutierrez, Silla‐Martinez, and Gabaldon 2009). Then, we concatenated the genes to a super matrix with FASconCAT‐G v1.0499 (Kuck and Longo 2014). Substitution models and optimal partitioning strategies for the 12 PCGs were conducted using the Bayesian Information Criterion in PartitionFinder v.2.1.1 (Kalyaanamoorthy et al. 2017; Appendix S3). Maximum likelihood phylogenetic trees with bootstrap replicates of 5000 were constructed using IQ‐TREE 1.6.10 (Nguyen et al. 2015). We employed the MCMCTree package within PAML v4.9j (Yang 2007) to estimate divergence times with the following parameter: 2 million generations with sampling every 10 generations after an initial burn‐in of 20,000 iterations. We conducted a comprehensive review of existing fossils to identify potential calibration points. Six spider fossils were included for temporal calibration (Table 1), and fossil selection was based on the study of Magalhaes et al. (2020). To ensure the reliability of our results, we conducted two independent MCMC analyses to confirm convergence. Finally, we utilized the ChiPlot online website (Xie et al. 2023) to visualize phylogenetic relationships and divergence times.

**TABLE 1:** Fossil information.

Groups	Fossils name	Minimum age (million years)	Maximum age (million years)
Mesothelae	*Palaeothele montceauensis* (Selden)	299	304
Mygalomorphae	*Rosamygale grauvogeli* Selden & Gall	242	247.2
Mygalomorphae	*Cretacattyma raveni* Eskov & Zonshtein	115	125
Synspermiata	*Montsecarachne amicorum* Selden	125	129.4
RTA	*Oxyopes succini* Wunderlich	43	47.8
RTA	*Almolinus ligula* Wunderlich	43	47.8

### 2.4 Biogeographical Reconstruction

To trace the biogeographic history of spiders, we used BIOGEOBEARS version v.1.1.2 (Matzke 2013) for ancestral region reconstruction. *A. bituberculata* was excluded from this analysis because they just inhabited the seafloor. Time‐calibrated phylogenetic trees were obtained using MCMCMCTree software in PAML (Yang 2007) based on Figure 4. We coded the geographic distributions of spiders on the basis of the World Spider Catalog (May 6, 2024) and recent studies (Ocque and Dippenaar‐Schoeman 2006; Ubick et al. 2005; Li and Lin 2016). The following realms were included: A, Palearctic realm; B, Nearctic realm; C, Ethiopian realm; D, Neotropical realm; E, Oriental realm; and F, Australian realm. We tested all the six models provided by BIOGEOBEARS: LAGRANGE's DEC, DIVALIKE, BAYAREALIKE, and respective models with the parameter +j. The models with +j allowed founder‐event speciation (Matzke 2014). In this study, each ancestor was allowed to occur in three or fewer regions. This option was selected because the maximum number of regions in which a spider can occur is 3 (Appendix S4). The resulting file from MCMCtree in PAML software was inputted to BGB for the selection of the best model. The Akaike weights of information criterion (AICc_wt) were used in comparing the six models. The likelihood ratio test (LRT) was used in testing the null hypothesis and determining whether a model has a likelihood value equal to the likelihood values of its +j models. A *p*‐value of < 0.05 for LRT indicates rejection of the null hypothesis.

### 2.5 Evolutionary Dynamics of Traits

Ancestral web types within Araneae were reconstructed using two applications: (1) RASP software (Yu, Blair, and He 2020) and (2) PastML (https://pastml.pasteur.fr/; Ishikawa et al. 2019). A marginal posterior probability approximation with an F81‐like model was used. For RASP, we selected three methods to estimate the distributional probability of ancestor nodes: statistical BioGeoBEARS package (S‐BGB; Matzke 2013, 2014), statistical dispersal‐vicariance analysis, and binary Bayesian MCMC (BBM). In the S‐BGB methods, we also tested all six models provided by BIOGEOBEARS. The most commonly used and complex F81 + Г model was used in BBM model analysis. The MCMC chains were run for 10,000,000 generations, four chains were run, the sampling frequency was 1000, and the starting 25% of the samples were discarded as aging samples. For the above analyses, the maximum ancestral trait of a node was limited to 4. Web trait data were primarily obtained from the World Spider Catalog (May 6, 2024) and recent studies (Ocque and Dippenaar‐Schoeman 2006), and are documented in Appendix S5. According to the presence or absence of webs and their shapes, the data were categorized into the following types: A, curtain/tunnel web; B, funnel web; C, irregular web; D, lampshade web; E, loose space web, F, mesh‐like web; G, orb web; H, purse web; I, scatter web; J, sheet web; K, silk‐lined tubular retreat; L, star‐shaped sheet web; M, trapdoor; N, free living, O, silken retreat; and P, cave dweller.

### 2.6 Evolutionary Rates and Positive Selection Analysis

To study variations in nucleotide substitution rates in the spider mitogenomes, we retrieved all 13 mitochondrial PCGs from the mitogenomes of 28 spiders (Appendix S6). These mitogenome PCGs were extracted using PhyloSuite (Zhang, Jiang, et al. 2020; Zhang, Gao, et al. 2020), and we subsequently used MAFFT (Katoh and Standley 2013) to compare each PCG with a codon‐based codon model. Ambiguous regions in each comparison were removed using Gblocks v0.91b (Castresana 2000). Finally, *Selenops bursarius* was defined as a reference for calculating the ratios of synonymous (dS) and nonsynonymous (dN) substitution rates were computed using KaKs_Calculator (Zhang et al. 2006) with the yn00 model. To assess selection pressure stemming from the extreme environment, we defined *D. jiaxiang* and *A. aquatica* as the foreground branches. The Codeml package in PAML v4.9j (Yang 2007) was used in identifying potential positively selected genes (PSGs). Calculations for *D. jiaxiang* and *A. aquatica* were performed independently. That is, *A. aquatica* was removed from the gene set when the PSGs for *D. jiaxiang* were calculated, and *D. jiaxiang* was removed from the gene set when the PSGs for *A. aquatica* were calculated. This procedure prevented interactions between the two spiders. For the results produced by the branch‐site model, raw *p*‐values (LRT) were corrected using the false discovery rate (FDR) method, and most genes would have been filtered out by an overly strict correction method. Instead of FDR, we used a compromise approach, where an LRT must produce a *p*‐value of less than 0.05, and at least one BEB site is present (Bayes empirical Bayes site, posterior probability > 0.5), and all BEB sites are contiguous.

### 2.7 Convergent Evolutionary Analysis

To increase the accuracy of the convergent evolutionary analysis, we included 28 spiders that were closely related to the two aquatic spiders and had good‐quality genome annotations (Appendix S6). Raw protein sequences were compared using PRANK (Loytynoja 2014), filtered with Gblocks (Castresana 2000), and converted into PAML format. Selective pressure analysis was then performed using the Codeml package of PAML software, and the model was model 0 (the one ratio model). In the generated rst file, the ancestral sequences of each node were extracted, in addition to the evolutionary tree comprising the evolutionary rate of each PCG. In this study, two methods were used in detecting convergent evolutionary events at the molecular level. The first was an amino acid substitution‐based method using convCal (Zou and Zhang 2015), and the second was the PCOC, which considers the properties of amino acids, such as molecular size and charge and polarity (Rey et al. 2018).

## 3 Results

### 3.1 Characterization of the Spider Mitogenomes

**FIGURE 1:**
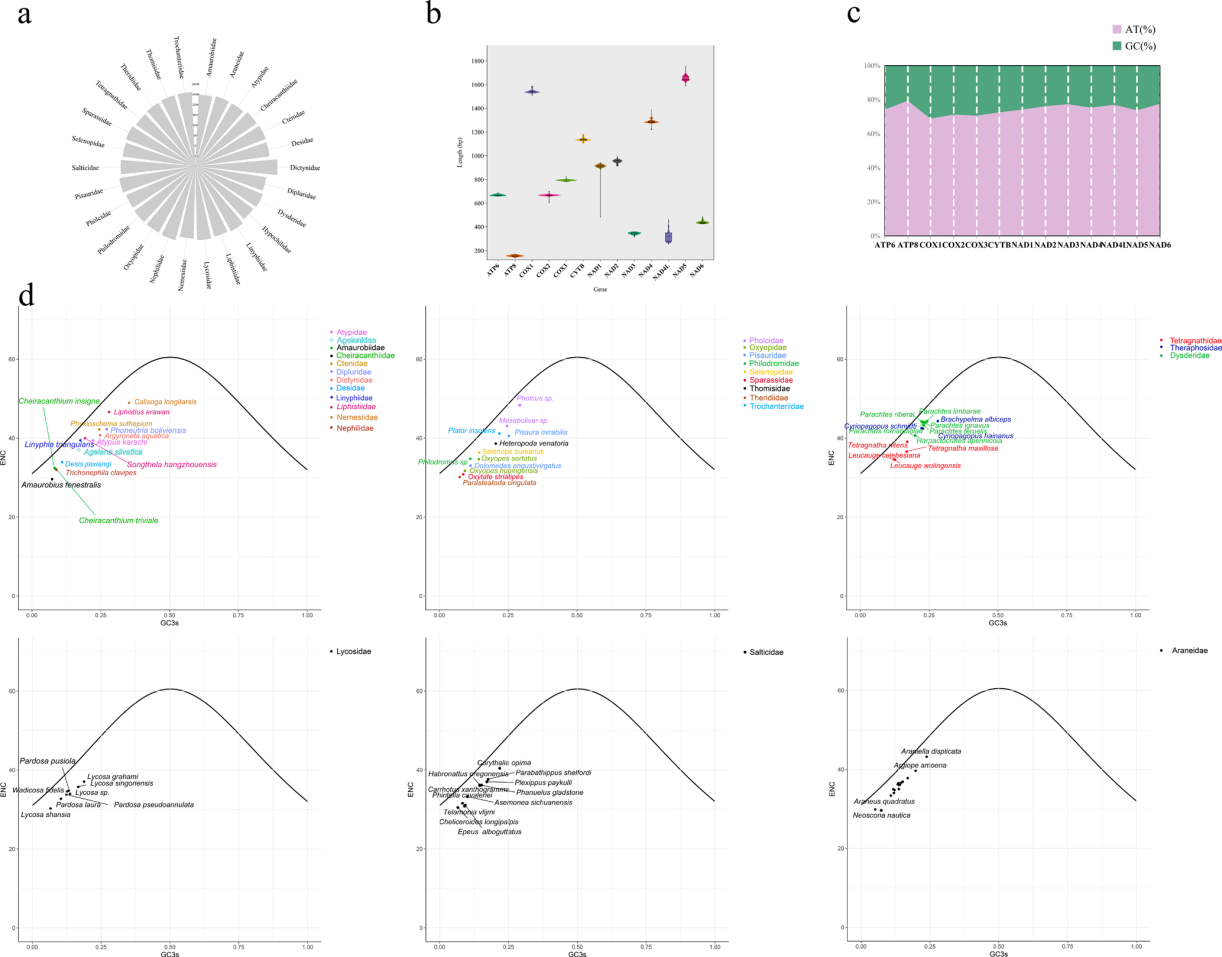
Characterization of the mitogenome of spiders. (a) Mean mitochondrial length in different families. (b) Average length of 13 mitochondrial PCGs. (c) Average AT/GC content of 13 mitochondrial PCGs. (d) ENC plotted against GC3s based on PCGs of 76 spiders. The solid line indicates the expected curve of positions of genes when the codon usage is merely determined by the GC3s composition.

After assembly and re‐annotation, we obtained 255 nearly complete spider mitogenomes. Given that some spider mitogenomes failed to loop, we selected only 76 mitogenomes with good annotation and complete loops for mitogenomes characterization. *Lycosa singoriensis* had the smallest mitogenome length (136,686 bp), whereas *A. aquatica* had the longest (16,000 bp; Appendix S7). At the family level, the longest average genome length was found in Dictynidae, and the smallest in Amaurobiidae (Figure 1a; Appendix S7). In addition, we measured the length of 13 PCGs from 76 species. *ND5* had the longest average length, followed by *COX1*, and *ATP8* had the shortest (Figure 1b; Appendix S7). The *ND5* and *COX1* genes encode the nicotinamide dinucleotide dehydrogenase (NADH) and cytochrome c oxidase subunits, respectively, which are widely used as molecular markers for species identification (Astrin, Huber, et al. 2006). The *COX1* gene had the lowest AT content (68.8%), whereas the *ATP8* gene had the highest (79.3%; Figure 1c).

### 3.2 Analyses of Codon Usage

We analyzed the codon usage frequency and RSCU ratios for 13 PCGs within the 76 spider mitogenomes. The distribution of the RSCU values is shown in Figure S1. In total, the genes in the 76 mitogenomes contained 277–17,860 codons (Appendix S8). Phenylalanine and isoleucine were the most prevalent amino acids in the 76 mitogenomes (Appendix S8). A total of 31 codons exhibited RSCU values greater than 1. The most pronounced codon preference was observed for UUA (RSCU ratio = 2.6804), followed by codon preference for CCU (RSCU ratio = 2.0837), which encoded proline and leucine, respectively (Figure S1; Appendix S8). The ENC values of the 13 PCGs in the 76 spider mitogenomes ranged from 23.98 (*ND3* of *Pisaura bicornis* ) to 60.11 (*ND4L* of *Carrhotus xanthogramma* ; Figure S1; Appendix S9). The majority of spiders exhibited mitochondrial PCGs with ENC values greater than 35, indicating a lack of strong codon usage preference (Appendix S9). Comparative analysis of codon usage across different spider families (excluding families with fewer than three species) revealed a consistent codon usage pattern in mitochondrial PCGs across species from different families (Figure S1). With the exception of the ATG and TGG codons, the RSCU values for all other codons were less than 1, suggesting that codon usage does not vary significantly at the family level (Appendix S10). Additionally, an analysis of nucleotide composition and codon usage in the mitochondrial PCGs showed that the ENC values of all species fell below the standard curve, and this trend was consistent across species from different families. The observed pattern of codon usage suggests that codon usage in spider mitotic genomes is primarily influenced by natural selection pressures rather than G/C mutation bias (Figure 1d).

### 3.3 Phylogenetic Results and Gene Arrangement

**FIGURE 2:**
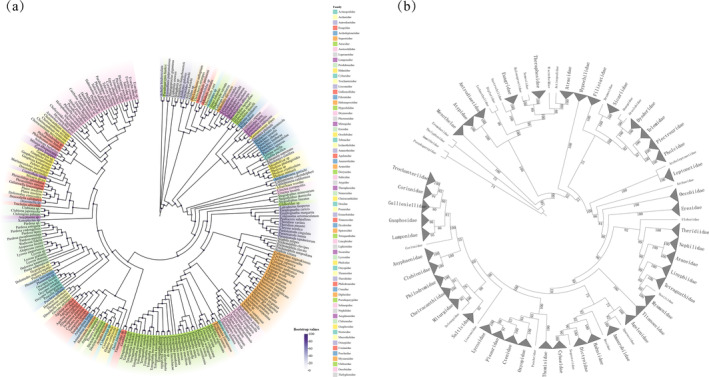
Results of phylogenetic analysis of spiders. (a) Phylogeny of spiders inferred from the 13 PCGs of the 255 spider mitogenomes was examined using maximum likelihood (ML) methods. (b) Topology among the 66 families of spiders.

**FIGURE 3:**
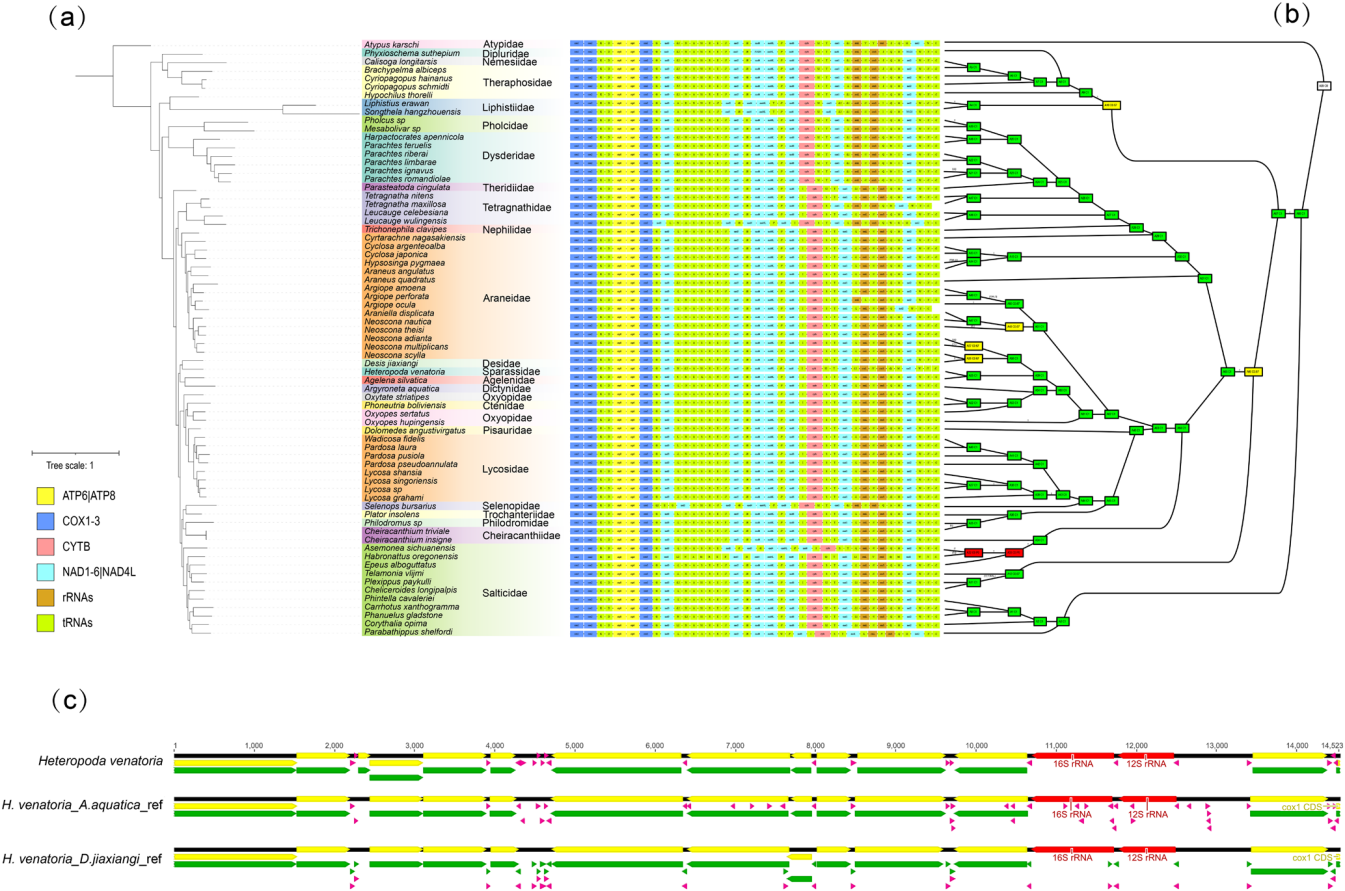
Gene rearrangement analysis. (a) Maximum likelihood phylogenetic tree inferred from 13 PCGs of 71 spider mitogenomes. (b) Representation of the output of the TreeREx analysis on maximum likelihood tree (ML‐1) with gene arrangement. The rearrangements on the branches are given as Transposition (T), Inversion (I), and Inverse transposition (iT). (c) Gene order of the mitogenome of *Heteropoda venatoria* when based on different references.

We generated a super matrix of 6608 loci using 12 PCGs from 255 spiders and 4 outgroups and constructed a phylogenetic tree with the maximum likelihood method (Figure 2). Our results were well supported at the higher orders of spiders, such as Mesothelae, Opisthothelae, Mygalomorphae, and Araneomorphae, in the Atypoidea, Avicularioidea, Synspermiata, Araneoidea, Marronoid clade, and Oval Calamistrum clades, and in Dionycha. Filistatidae is a sister group to Synspermiata, and the two clades form a sister clade to Hypochilidae. This finding is inconsistent with the findings of previous studies, which suggested that Filistatidae and Hypochilidae are sister groups. Our data confirmed that Leptonetidae is monophyletic and sister to the remaining clades. The general family of Araneoidae is well clustered, but internal relationships are problematic. For example, Titanoecidae stands alone as a clade, Dictynidae under the marronoid clade is non‐monophyletic, and its topology results are as follows: *A. aquatica* , *Pisaura mirabilis* , *Dictyna arundinacea* , *Dictyna namulinensis* . We identified a total of 346 cases of translocation events, 267 cases of Inversion events, 60 cases of tandem duplication and random loss events and 89 cases of inverse transposition events (Figure 3; Appendix S11). The gene order of the different spiders are mostly similar to each other, and few translocations and inversions were detected compared to the putative ancestral gene order.

### 3.4 Divergence Time

**FIGURE 4:**
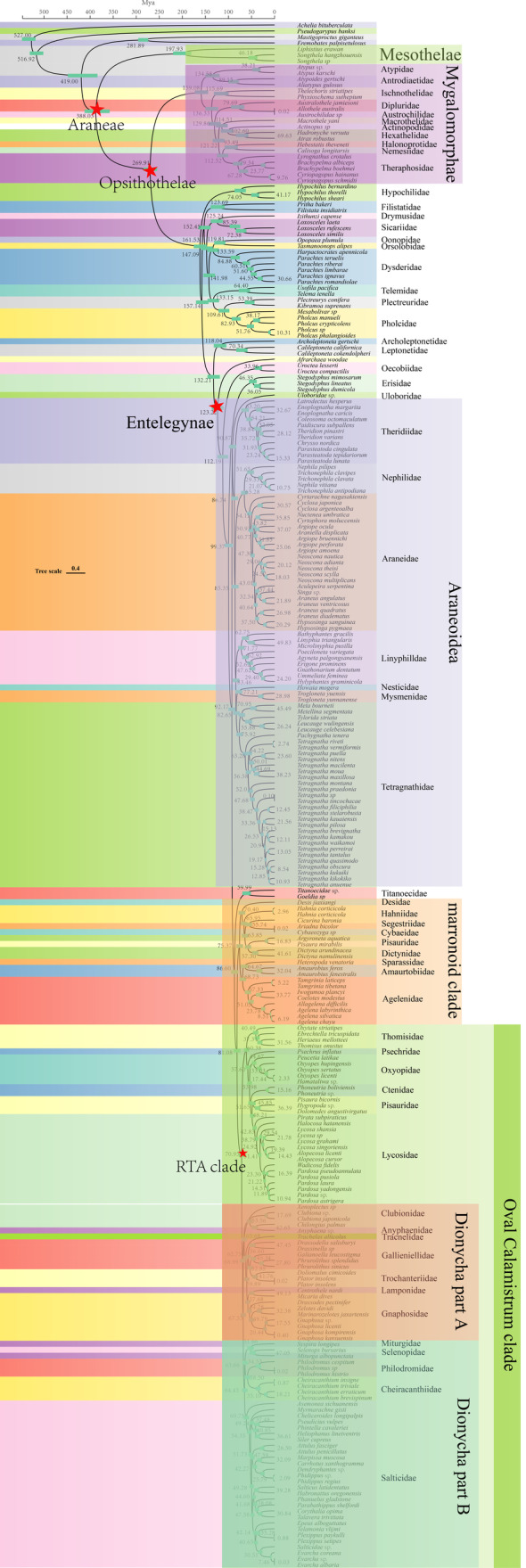
Estimates of the divergence time of spiders were inferred from an analysis of 259 complete mitogenomes using Bayesian MCMCTree with the independent‐rates relaxed‐clock model. Values are shown next to nodes with mean estimates and 95% confidence intervals.

We performed CDS sequence concatenation on 13 PCGs, which we trimmed to obtain a super matrix dataset. The mcmctree program in paml v4.9 was used in estimating the divergence time of spiders. The topology of the species tree used is shown in Figure 2a. Fossil calibration analyses have shown that the missing gene data model are robust (Evangelista et al. 2019). Our divergence time results showed that the origin of spiders dates from the early Devonian (~416.92 mya [95% PHD: 373.65–382.08; Figure 4]), the separation of the two clades of Mesothelae and Opisthothelae from the late Carboniferous occurred approximately 301.71 mya (95% PHD. 299.08–304.06), the time of the separation of Atypoidea with Avicularioidea was approximately 243.95 mya (95% PHD: 241.81–246.89) in the middle Triassic, the time of origin of the Araneomorphae was approximately 256.73 mya (95% PHD: 246.71–267.29), and the divergence time of the Araneomorphae was approximately 212.95 mya (95% PHD: 194.95–233.31) in the Late Triassic. The most primitive clade of Araneomorphae is the Hypochilidae, which originated in the Early Jurassic 179.02 mya (95% PHD: 167.84–192.35) and differentiated internally in the Early Cretaceous (ca. 118.91 mya; 95% PHD: 81.26–160.8). Filistatidae diverged from the Synspermiata clade in the mid‐Jurassic at approximately 168.92 mya (95% PHD: 163.85–174.64), the internal divergence of Synspermiata occurred in the Early Cretaceous at approximately 127.54 mya (95% PHD: 125.17–129.51). Moreover, Palpimanoidea separated from Entelegynae at approximately 174.86 mya (95% PHD: 170.26–179.57), and internal differentiation occurred at approximately 169.88 mya (95% PHD: 166.07–174.38) in the Middle Jurassic. Araneoidea separated from the rest of the taxa in the Early Cretaceous approximately 130.14 mya (95% PHD: 125.02–134.96), the marronoid clade with the RTA branch is around 108.1 mya (95% PHD: 97.57–119.21). The divergence of the Oval Calamistrum clade with the Dionycha clade occurred in the Late Cretaceous period approximately 91.22 mya (95% PHD: 82.69–99.35), and the divergence time of the Dionycha with the Prodidomidae occurred approximately 86.36 mya (95% PHD: 78.5–95.09).

### 3.5 ANI Analysis

Owing to the specific habitat conditions under which *A. aquatica* and D. *jiaxiangi* live, we selected 26 closely related species in 14 families in ANI analysis (Figure S3b; Appendix S12). *A. aquatica* was closely related to *P. mirabilis* , with 99.12% sequence similarity (Figure S3b; Appendix S13). *D. jiaxiangi* shared 83.48% sequence identity with *Argiope ocula* (Figure S3b; Appendix S7). *A. aquatica* and *D. jiaxiangi* exhibited a sequence similarity of 77.69%. In addition, although Araneidae, Lycosidae, and Salticidae are distantly related, we noted high ANI values in the three species. Interestingly, high ANI values were observed in spiders from different families, facilitating spider identification. The high ANI values may be useful in the identification of spider species and evolutionary studies.

### 3.6 Ancestral Range Dispersal and Trait Evolution

**FIGURE 5:**
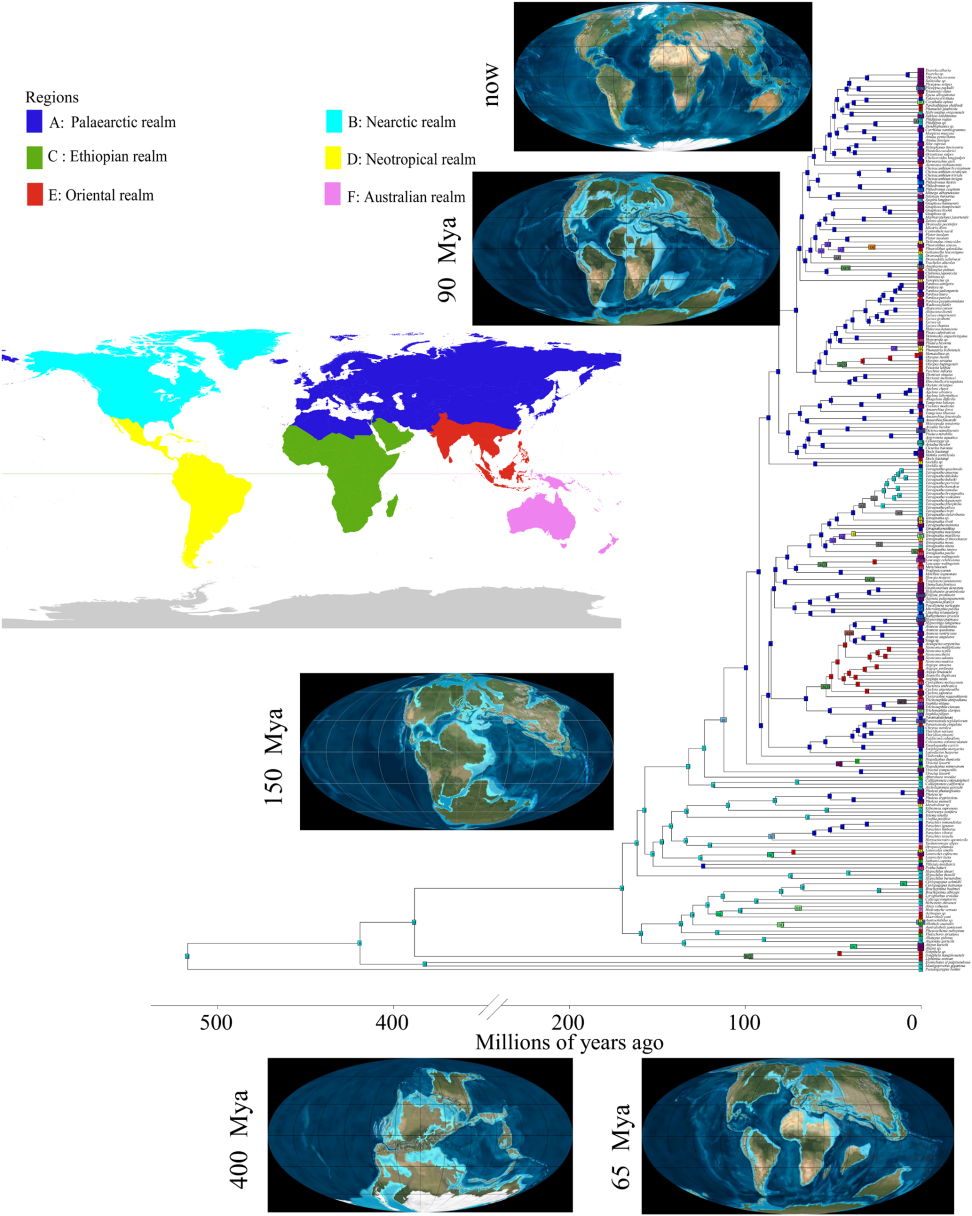
Estimating ancestral ranges of Zingiberaceae species using BioGeoBEARS and BAYAREALIKE+j model.

**FIGURE 6:**
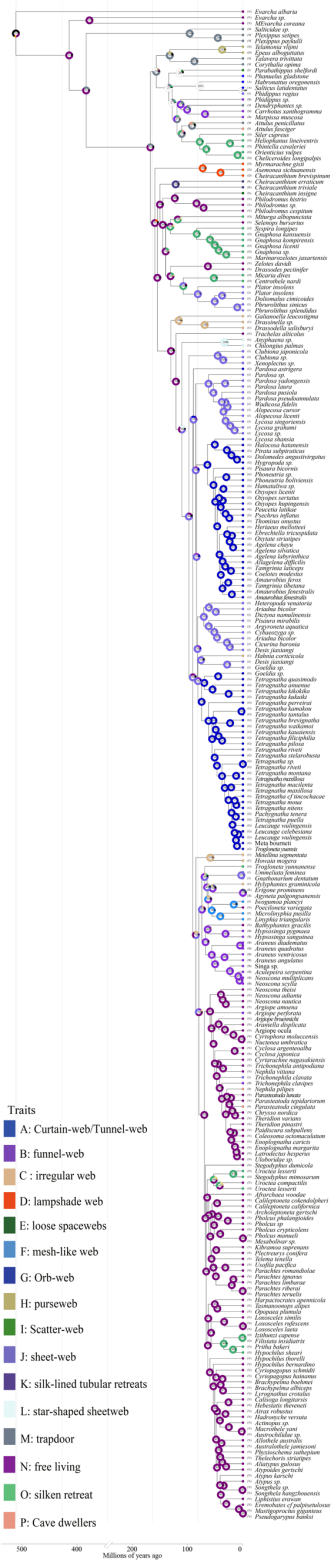
Ancestral trait reconstruction of web type in spiders using RASP.

The corrected Akaike information criterion model selection supported the BAYAREALIKE+j model, although the DEG+j model provided similar results in model selection and ancestral area reconstruction (Figure S4; Appendix S14). The estimated ancestral regions suggested that the ancestor of spiders probably originated in the Devonian Nearctic realm and then spread to the Palaearctic and Oriental realms (now Europe and Asia, respectively; Figure 5). The ancestral trait was reconstructed using RASP (Figure 6; Appendix S15) and PastML (Figure S5; Appendix S16) for web types across the spider phylogeny. The results suggested that the common ancestor of spiders may have been free living (92.66%; Figure 6; Appendix S15). The common ancestor of Mesothelae and Mygalomorphae maintained free‐living (62.22%) or trapdoor habits (33.29%; Figure 6). The ancestor of Araneomorphae showed the lampshade web trait (13.57%) in addition to maintaining free‐living habits (76.90%). The lampshade web trait was 35% in the pastml results (Figure S5; Appendix S16). The most primitive taxon of the Araneomorphae (Hypochilidae) maintained the lampshade web. The emergence of orb webs seemed to be traced back to the araneoid ancestors. The ancestors of the evolutionary branch of the RTA did not build foraging webs but evolved into the free‐living mode after losing the ability to weave webs. Notably, sheet webs evolved independently in several taxa in the Araneomorphae, including Pholcidae, Eresidae, Theridiidae, Linyphiidae, and Hahniidae.

### 3.7 Selection Analyses

Owing to the broad distribution patterns of spider species, we argued that spider mitogenomes are subject to different environmental selection pressures in different environments. For example, the only *A. aquatica* that live in freshwater and the *D. jiaxiangi* that live in tidelands are necessarily subject to greater selection pressures to adapt to extreme environments. Therefore, we selected 28 spider species that are closely related to *A. aquatica* and *D. jiaxiangi* for selection pressure analysis. First, we selected *S. bursarius* as a reference and the yn00 module of PAML v4.9j software (Yang 2007) was used in calculating the pairwise NSRs of the 13 mitochondrial PCGs of the 28 spiders, including the dN value, dS value, and the ratio of dN to dS. However, most *K*_a_/*K*_s_ ratios of the 13 PCGs from 28 mitogenomes were less than 1. Only four genes had *K*_a_/*K*_s_ ratios greater than 1, and they were *ATP8* (*D. jiaxiangi*), *ND2* ( *Cheiracanthium triviale* ), *ND4* ( *Neoscona multiplicans* ), and *ND6* ( *Neoscona scylla* ; Appendix S17). Notably, *ATP8*, *ND2*, and *ND6* had the highest median *K*_a_/*K*_s_ value in the spider mitogenomes. Conversely, *COX1* and *ND1* had the lowest median *K*_a_/*K*_s_ value. In addition, *Argiope amoena* , *Argiope perforata* , and *Pardosa laura* had the highest median *K*_a_/*K*_s_ value. *Neoscona nautica* had the lowest median *K*_a_/*K*_s_ value.

**FIGURE 7:**
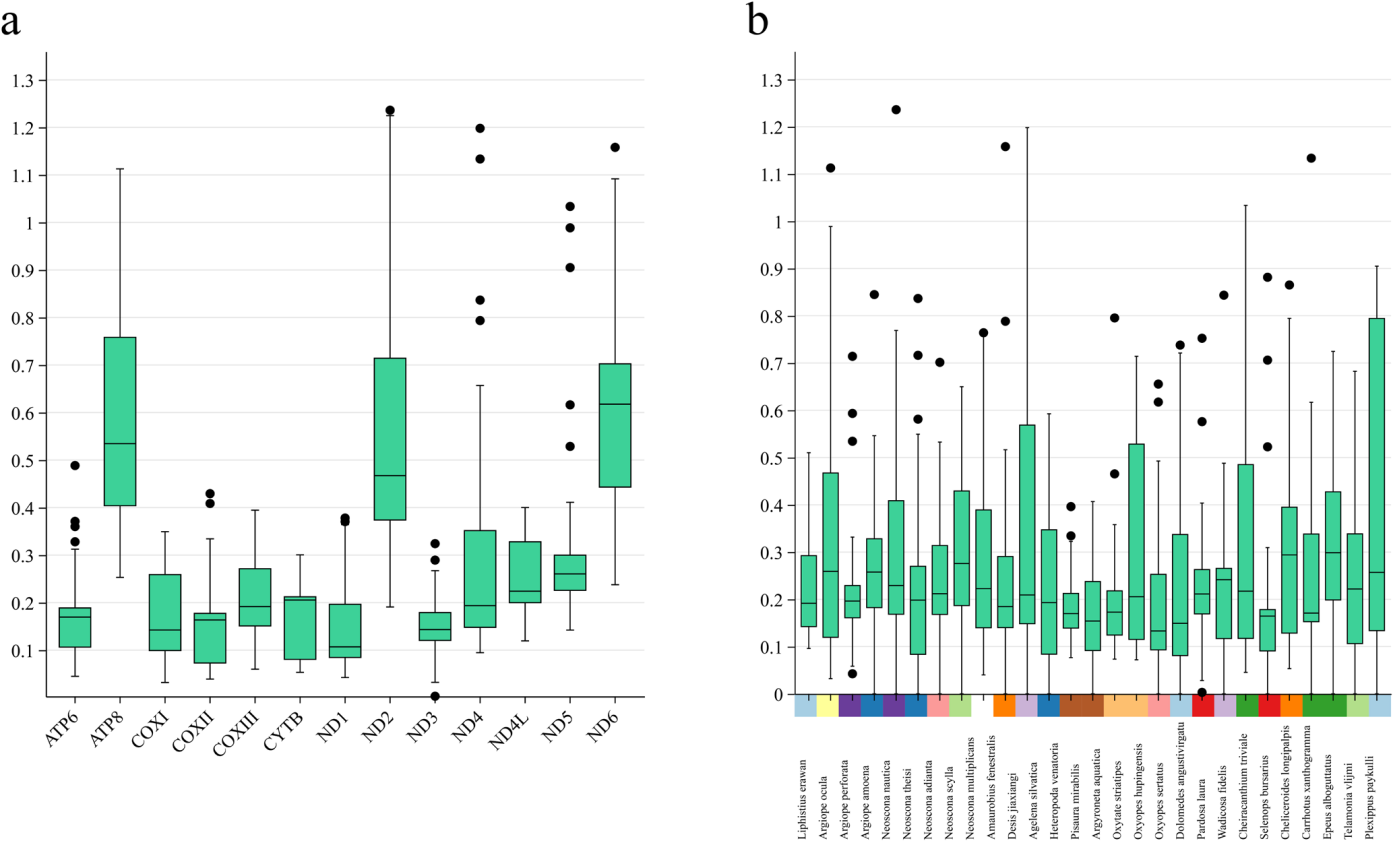
Boxplots of *K*_a_/*K*_s_ for the (a) 13 mitochondrial PCGs and (b) 23 spider mitogenomes.

Positive selection signals were often considered the imprint of species' adaptation to their environment (Nielsen 2005). To identify all putative PSGs and REGs in *A. aquatica* and *D. jiaxiangi*, we generated datasets of 13 PCGs from 28 spider species. The topology of the species tree used is shown in Figure 7a. When branch and branch‐site model tests were performed on *A. aquatica* , *D. jiaxiangi* was removed from the background set of taxa. *A. aquatica* was removed from the background set of taxa. Finally, we identified three and two REGs in *D. jiaxiangi* and *A. aquatica* , respectively, using the branch‐site model (Table 2). One PSG was identified in *D. jiaxiangi* but not in *A. aquatica* with the branch‐site model (Table 3). Then, the evolution rate (d*N*/d*S*) of each clade of the REG (*ATP6*) was calculated using PAML. The LRT showed that the free ratio model considerably outperformed the one ratio model. We found that the *ATP6* gene occurred on at least two evolutionary branches. The first accelerated evolution occurred in the ancestral evolutionary clade of *Amaurobius fenestralis* and *D. jiaxiangi* (ω = 0.0560128). The second accelerated evolution occurred in the clade of *D. jiaxiangi* with an ω value of 0.124 (Figure S6).

**TABLE 2:** Positively selected genes and sites detected in the mitogenomes of *Desis jiaxiangi*.

Species	ID	−ln *L* (null model)	−ln *L* (alternative model)	LRT	*p*	BEB sites
*D. jiaxiangi*	ATP8	−254.887943	−241.753214	26.269458	0.000000297	—

**TABLE 3:** Rapidly evolving genes detected in the mitogenomes of *Desis jiaxiangi* and *Argyroneta aquatica* .

Species	ID	−ln *L* (one ratio)	−ln *L* (two ratio)	LRT	*w* (background)	*w* (foreground)	*p*
*D. jiaxiangi*	ATP6	−8916.499076	−8914.40755	4.183052	0.04342	0.0995683	0.040830111
*D. jiaxiangi*	ND1	−5689.628377	−5687.675897	3.90496	0.0249269	0.0717557	0.048143774
*A. aquatica*	ND3	−3378.655179	−3375.636011	6.038336	0.0233707	NA	0.013998473
*A. aquatica*	ND4L	−2445.144098	−2441.745786	6.796624	0.332869	0.0001	0.009133041
*A. aquatica*	ND5	−21218.34308	−21215.21635	6.253462	0.0386351	0.148356	0.012395082

*Note:* Values represent the non‐synonymous substitution rate/synonymous substitution rate (d*N*/d*S*), which is calculated by the free ratio model. NA represents the case where the d*N*/d*S* value is 999 when the dS is zero.

### 3.8 Convergence of Adaptations

**FIGURE 8:**
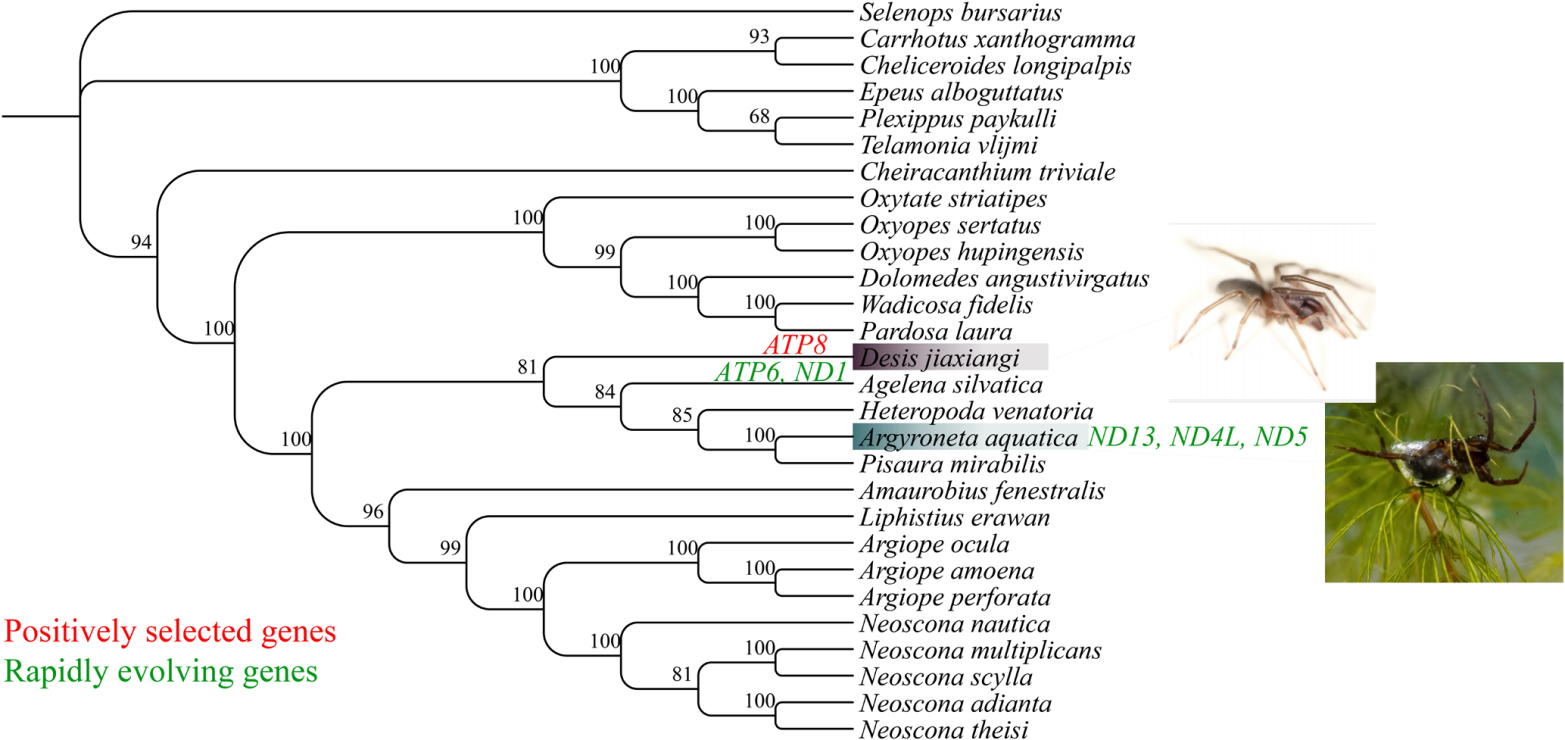
Phylogenetic relationship of spiders used in tests for selection. Rapidly evolving genes are marked green, and positively selected genes are marked red.

We speculated whether both aquatic spiders developed convergent adaptation to aquatic environments. To test for convergence, we generated one data set containing 28 spiders. We used the JTT‐Fgene model (Zou and Zhang 2015) and compared the observed number of convergent amino acid substitutions between *A. aquatica* and *D. jiaxiangi* with the neutral expectations and identified four genes that are under convergent evolution (Appendix S18). Using the PCOC method (Rey et al. 2018), which considers shifts in amino acid preference instead of convergent substitutions, we found three genes that have undergone convergent evolution (Appendix S18). One gene (*ND5*) was identified by both methods (Figure 8).

## 4 Discussion

### 4.1 Mitogenome Characterization

Spider mitogenomes differ in AT content in the coding region from 68.8% to 79.3%. The mitogenomes of *Escherichia coli* and *Plasmodium falciparum* have abnormally high AT content (Hamilton et al. 2016), and the recombination rate and AT content showed an opposite correlation in mammalian and other animal genomes (Lattorff et al. 2007). In some arthropods, high AT content may be the result of reduced genome size, virtually absent DNA methylation, and age‐specific energy requirements (Dennis et al. 2020). Furthermore, most PCGs in the mitogenomes were below the expected ENC curve, indicating that natural selection is the main factor shaping codon usage preferences (Zhang, Jiang, et al. 2020; Zhang, Gao, et al. 2020). This finding is particularly true for ATP‐ and NADH‐related genes, which were subject to strong environmental selection pressure. However, not all ATP‐ and NADH‐related genes showed ENC values that fell on the expected curve, suggesting that mutations played a minor role in shaping codon preferences. These findings suggested that natural selection is the primary factor driving codon usage preferences in spiders, particularly for important genes, such as those involved in ATP and NADH production.

Previous studies have suggested that rearrangements in spider mitogenomes may often be due to annotation errors. For example, the mitogenome of *A. fenestralis* (Amaurobiidae) has the highest number of annotation errors, reporting *trnS*, *trnR*, *trnE*, ‐*trnL*, and ‐*trnF* genes, but omitting annotations for the *trnA* and *trnN* genes (Prada et al. 2023). In this work, we observed a similar issue, when annotating spider mitogenomes, the annotation results (including the number of genes and gene order) varied depending on the reference mitogenome used (Figure 3c). Specifically, when annotating the mitogenome of *Heteropoda venatoria* using the mitogenomes of *D. jiaxiangi* and *A. aquatica* as references, we found that while 13PCGs were annotated correctly, there were discrepancies in the number and positioning of tRNAs. These findings align with those of a previous study (Tyagi et al. 2020; Prada et al. 2023; Ma et al. 2023). Our results further confirmed that annotation errors in spider mitogenomes are primarily concentrated in tRNA genes. However, misannotations are not unique to spiders, errors in organelle genome annotations are a common phenomenon across species. Because current phylogenetic studies primarily rely on PCGs or sequences of PCGs+ non‐coding RNAs (ncRNAs), which do not require a precise alignment of all genes. As a result, annotation errors are inevitable, especially when mitochondrial data are generated by different researchers or derived from different species with varying evolutionary relationships. For these reasons, we chose not to delve further into the issue of gene rearrangements in this study. As concluded by Ban, Shao, and Wu (2022) due to the current deficit in the number of spider mitogenomes, most of which were mites and ticks, more mitogenomes of non‐Acari orders are warranted for future sequencing to refine the rearranged mitogenomes. Meanwhile, to resolve these annotation errors in spider mitogenomes, a comprehensive and systematic reannotation effort is required. While this process would be labor‐intensive, it is necessary to achieve more accurate and reliable mitogenomic data.

### 4.2 Phylogenetic Relationships of Spiders

Our results restored the monophyly of Mygalomorphae and Araneomorphae (BS = 99). Furthermore, our results are consistent with previous studies supporting the idea that Mygalomorphae consists of two subfamilies (Avicularioidea and Atypoidea; BS = 100; Hedin et al. 2019; Kallal et al. 2021). Araneomorphae is a well‐established clade and is strongly supported in all morphological and molecular phylogenies (Bond et al. 2014; Kulkarni et al. 2021; Ramirez et al. 2021; Kallal et al. 2021). As the most primitive taxon in Araneomorphae, Hypochilae was designated as the sister taxon of Filistatidae in terms of morphology. Meanwhile, transcriptome analysis by Bond et al. (2014) showed that Filistatidae is the sister taxon of Hypochilae. Wheeler et al. (2017) discovered the Hypochilidae + Filistatidae group (with only 60% support for the root), and the representative species of Filistatidae, *Prithinae* sp. Costa Rica MR11, clustered into Dysderidae. These results suggested that the phylogenetic relationships were not robust enough. However, in our research, we showed that Filistatidae clustered into a single taxon with Synspermiata, which comprised the monophyletic taxa Dysderoidea (Drymusidae + Scytodidae + Sicariidae) and Scytodoidea (Dysderidae + Oonopidae + Orsolobidae + Segestriidae) and tracheal loss clades (Pholcoidea and Caponioidea). Although previous studies supported the monophyly of Synspermiata and their sister taxon grouping with Hypochilae + Filistatidae (Wheeler et al. 2017; Garrison et al. 2016; Fernandez et al. 2018; Kulkarni et al. 2021; Ramirez et al. 2021), the small amount of data on spiders involved in these studies largely affected the phylogenetic relationships. In our study, the use of a larger number of species largely improved phylogenetic stability. Thus, our results supported Hypochilae as the most primitive monophyletic taxon of Araneomorphae (83% support for the root node).

Ledford and Charles (2010) showed that Leptonetidae is non‐monophyletic. The studies of Wheeler et al. (2017) and Ramirez et al. (2021) supported this result. Garrison et al. (2016) considered Leptonetidae the sister group of Entelegynae. Our phylogenetic results also supported the non‐monophyly of Leptonetidae, which formed a sister–clade relationship with Archoleptonetidae. The two families formed a sister clade with Araneomorphae (BS = 100). Entelegynae was treated as the sister taxon of Palpimanoidea, and previous findings supported the monophyly of Entelegynae (Garrison et al. 2016; Wheeler et al. 2017; Fernandez et al. 2018; Kulkarni et al. 2021; Kallal et al. 2021). This result is consistent with the results of the present study. Finally, Anyphaenidae was currently classified under Dictynoidea. However, results showed that it was not clustered with the other species of Dictynoidea (BS = 100). Therefore, further confirmation is needed as to whether Anyphaenidae belongs to Dictynoidea. Although Wheeler et al. (2017) reclassified all families in the marronoid families, their results have not been well supported (only 28% support for root node and a minimum of 2% support for internal nodes); moreover, the results did not indicate Sparassidae as a sister clade of Amaurobiidae, and Sparassidae was removed in constraint analysis. Thus, the placement of Sparassidae remains unresolved. Our results reclassified marronoid families with good support (75% support for root nodes and a minimum of 73% support for internal nodes) and strongly supported Sparassidae as a sister clade of Amaurobiidae. Moreover, Wheeler et al. (2017) indicated that Psechridae has close relationships, is unusually related to Thomisidae, and has only 25% support for being monophyletic. Our results demonstrated 95% support for Psechridae as monophyletic. Wheeler et al. (2017) divided Ctenidae into two clades. One of the clades was a sister clade to Lycosidea; and the other, with Psechridae + Lycosidae. Their results did not resolve the positional relationships of Ctenidae. Our study supported Ctenidae as the sister clade of Lycosidae + Pisauridae. In summary, increases in the number of species and quality of sequencing increases (from gene markers to complete mitogenomes) facilitates studies on the phylogenetic relationships of the spider order.

### 4.3 Divergence Time Estimation

An increase in the number of fossils we calibrated resulted in a large mean age estimate for the root taxa of Araneae. The estimate was larger than the estimates in most previous studies. For example, our estimated mean age of divergence for Araneae was 388.03 mya (Figure 4), which is ~59 mya earlier than the result of Li et al. (2022) and ~40 mya earlier than the estimates of Shao and Li (2018) and Garrison et al. (2016). However, it is approximately 8 mya later than that calculated by Wang et al. (2022), who used the nuclear genome. In addition, our results are similar to those of previous studies in most of the major evolutionary clades. For instance, our results indicated that the divergence of Mygalomorphae and Araneomorphae occurred at approximately 269.91 mya, similar to the results of Li et al. (2022) (258 mya). Differences in the results may be due to variations in sampling taxa, molecular phylogenies, and fossils among the studies. Spiders have undergone approximately 400 mya of evolution (Garrison et al. 2016; Magalhaes et al. 2020; Selden et al. 1996), and major historical events (e.g., changes in geology and climate) during this period may have affected the spread and evolution of spiders. However, age estimates for most major evolutionary clades coincide with their first appearance in the fossil record: Mygalomorphae (Triassic fossils), Synspermiata (Jurassic fossils), Araneoidea (Cretaceous), and RTA clade (Jurassic fossils; Shao and Li 2018; Magalhaes et al. 2020; Li et al. 2021, 2022). In addition, we found that the three major clades of RTA‐clade spiders already appeared in the Cretaceous, and the first evolutionary clade is the marronoid clade, which appeared approximately 81.08 mya. The Oval Calamistrum clade appeared approximately 70.95 mya, and Dionycha appeared approximately 68.99 mya. Thus, RTA‐clade spiders may have originated in the Mesozoic (Magalhaes et al. 2020; Garrison et al. 2016; Fernandez et al. 2018). Notably, our estimates of the divergence time of the RTA clade were somewhat later than those estimated by Magalhaes et al. (2020). However, after they increased the maximum constraint value of the RTA clade at 99.41 mya, all node ages became younger, especially within the RTA clade, with the divergence time of the marronoid clade becoming 82 mya, the Oval Calamistrum clade becoming 80 mya divergent, and Dionycha at approximately 76 mya. Based on the available fossil records, 17 families of spider are present in the Cretaceous (Antrodiaetidae, Archaeidae, Hersiliidae, Ochyroceratidae, Oecobiidae, Oonopidae, Pacullidae, Palpimanidae, Telemidae, Tetrablemmidae, Theridiidae, Theridiosomatidae, Liphistiidae, Dipluridae, Leptonetidae, Mecysmaucheniidae, and Uloboridae), demonstrating that 41 lineages of spiders (approximately 35% of the available family lineages) crossed the K‐Pg boundary (Magalhaes et al. 2020). Hypochilidae is currently known worldwide to have two genera (*Hypochilus* and *Ectatosticta*) and 33 species. *Hypochilus is* distributed only in the United States, whereas *Ectatosticta* is distributed only in China. The distribution pattern between Eurasia (Old World) and North America (New World) is intermittent, similar to the distribution pattern of many plants and animals (Wen 1999; Sanmartin, Enghof, and Ronquist 2001; Brant and Jason 2007). Biogeographers believe that the Trans‐Cretaceous land bridge formed from the Mid‐Cretaceous to the Late Cretaceous (112–125 Ma) and played an essential role in biological exchange (Jiang et al. 2019). The results of the present study indicated that the internal divergence time of *Hypochilus* was 74.05 mya, but a recent study showed that the divergence time of Ectatosticta and Synspermiata was 204.96 mya (Fan et al. 2023). Therefore, we hypothesized that the ancestor of Ectatosticta dispersed from the Trans‐Cretaceous land bridge and gave rise to the intermittent distribution pattern of the present genus and that the ancestor of Ectatosticta was dispersed from the Beringia. To confirm our hypotheses, biogeographic studies, such as ancestral range reconstruction, are needed. In addition, for the Araneidae, which has a high diversification rate, we find that rapid dispersal occurred between 40 and 50 mya. The Cenozoic resulted in an extremely hot event in the Paleocene–Eocene because of the large amounts of CO injected into the sea vapor system (Mclnerney and Wing 2011; Chen and Ding 2011). The temperature of the Earth rose to a maximum of approximately 50 mya and then began to cool steadily. Then at approximately 33.9 Ma, the temperature plummeted and the Eocene–Oligocene extinction event occurred. Therefore, the rapid differentiation of the family Eocene during this period may have been a response to severe climatic conditions. Subsequently, until the Oligocene, temperatures increased again, in a period known as the Oligocene–Miocene Transition (OMT; Zhao and Li 2019). In this period, another rapid differentiation of Araneidae occurred. Around the time of OMT (25–22 mya), the global temperature was in a trough period. This phenomenon was accompanied by the onset of the rapid uplift of the Himalayas (Ding et al. 2017; Ma et al. 2017) and the lateral sliding of the Central South Peninsula (Zhang et al. 2024; Cao et al. 2011; Leloup et al. 1995), which may have been the main cause of this divergence. These events affected the differentiation of easterly zones, such as *Paini* (Che et al. 2010) and *Roscoea* (Zhao et al. 2016).

### 4.4 Ancestral Range Dispersal

Geographical distribution patterns based on extant species supported a Nearctic realm origin for spiders. For many years, fossilized arachnids have been found in the Upper Carboniferous coal systems of Europe and North America (Corda 1835; Buckland 1837; Meek and Worthen 1865). Uraraneida was once considered the closest relative of Araneae, and only two species of two genera have been found in its current position. *Attercopus fibriunguis* was found in Devonian fossils (390 mya) from Gilboa, New York (Selden, Shear, and Sutton 2008), and *Permarachne novokshonovi* was excavated in Permian fossils (270 mya) from Urals, Russia (Eskov and Selden 2005). Russell, Jason, and Paul (2016) described a new fossil arachnid, *Idmonarachne brasieri* gen. et sp. nov. from the Late Carboniferous (Stephanian, ca. 305–299 mya) in Montceau‐les‐Mines, France. A recent study unearthed a Late Carboniferous (310 mya) spider (Arachnida: Araneae) named *Arthrolycosa wolterbeeki* sp. nov. in Lower Saxony, Germany (Dunlop 2023). In addition, Shear et al. (1989) found a nearly complete spider spinneret in Devonian fossils in New York. This spinneret is the earliest fossil evidence of Araneae and the emergence of spinneret to date. On the basis of this ancient fossil evidence and our result, we speculated that spiders and their relatives first appeared in the Devonian Nearctic realm (now North America) and subsequently spread to the Palaearctic and Oriental realms (now Europe and Asia, respectively).

The genus *Tetragnatha* gradually spread from the Palaearctic realm to the Neopelagic and Neotropical realms approximately 50 mya. This data strongly support a direct terrestrial connection between Asia and North America. The common ancestor of *Tetragnatha* must have extended its range from eastern Asia to western North America via the Beringia. This conclusion is the same as that of a previous study on *Antrodiaetus* (Brant and Jason 2007). This extension likely occurred during the Eocene (53–36 mya), when the climate of Beringia was much warmer and wetter than it is today. During this period, a continuous band of deciduous hardwood and some tropical evergreens existed on Beringia (Wolfe 1975, 1978). This land bridge is extremely important to the dispersal of taxa that have adapted to warm temperate climates (Tiffney 1985). The Palaearctic and Neopelagics have a mixed fauna because of the connection of the Bering Strait in the past, and this connection has resulted in the numerous common characteristics of the current fauna in the holarctic, such as a number of shared species of birds and animals, including Moleidae, Ochotonidae, Castoridae, Gaviidae, Tetraonidae, and Acipenseridae. As a result, the Palaearctic and Neopelagic realms are also referred to as the holarctic. In conclusion, our results and fossil evidence support that the ancestor of spiders originated in the Neopelagic realm and subsequently spread to the Palaearctic and Neotropical realms and the presence of Beringia played an important role in the history of animal dispersal.

### 4.5 Evolutionary Dynamics of Traits

Spiders appeared after 400 mya and are the most diverse predators on Earth (Sensenig, Agnarsson, and Blackledge 2010; Huang et al. 2018; Magalhaes et al. 2020). Spiders possess up to eight different silk glands, which produce various types of silk (Garb 2013; Sensenig, Agnarsson, and Blackledge 2010; Fan et al. 2020). Spiders can be categorized into two groups according to their lifestyle: wandering and web‐building spiders (Foelix 2011). The former generally do not produce webs for hunting. They take an active approach and use spider silk for various purposes, including building burrows and wrapping their eggs (Foelix 2011; Vollrath and Selden 2007). This class of spiders consists primarily of most members of the evolutionary branch of the RTA. The latter uses spider webs for hunting in addition to basic functions, such as burrow building. Foraging behavior (i.e., based on the use of foraging webs or active hunting) has been linked to the patterns of diversity in spiders, and broad diversification rates have been found in Araneidae and RTA clade (Bond and Opell 1998; Blackledge et al. 2009; Dimitrov et al. 2012; Garrison et al. 2016). This result suggests that key innovations in spider silk use and web structure may not be the most dominant or only factors driving diversification. Our findings support the idea that the ancestors of spiders lived trapdoor lives. They would have used webs as linings for burrows and doors or as trip lines extending from the mouths of burrows. A recent fossil study of a nearly complete spider spinneret (Devonian) suggests that spiders evolved spinnerets similar to those of the extant spiders of Mesothelae in the Devonian or even before. This is the earliest evidence discovered for silk production from opisthosomal spigots and therefore for spiders. A report of an archaeognath insect found in the Early Devonian (Amesian; Labandeira, Beall, and Hueber 1988) and similar material found in the Late Devonian (Gietian) Gilboa fauna (Shear et al. 1984) establish an early origin for insects. However, none of these insects had wings. Therefore, we speculate that spiders of the Devonian period may have been tunnel‐ or tube‐dwelling predators. In addition, growing evidence has shown that orb spiders are not monophyletic (Dimitrov et al. 2012; Bond et al. 2014; Wheeler et al. 2017), raising the question of the possible multiple origins of orb webs. The results of the ancestral web architecture of the present study are consistent with the results of Fernaendez et al., which neither support a single orb web origin, as inferred in previous studies (Dimitrov et al. 2012; Garrison et al. 2016; Figure 6; Figure S5). Thus, neither orb weaving nor spinning can be traced to a single origin. The additional mitochondrial genomic data and the considerable increase in the number of taxonomic units may be the reason that the hypothesis of a single origin of orb weavers (called “ancient orb weavers hypothesis”) is not valid. Interestingly, ancestral range reconstruction showed that Araneidae species, which have a high rate of diversification, and their ancestors were mainly found in the Oriental realm. This region has a predominantly tropical rainforest climate with high biodiversity and high humidity. Thus, the evolution of orb weavers has enabled them to keep them off the ground and enhanced their hunting capabilities, serving as a key factor for their successful adaptation to humid tropical rainforest environments. Furthermore, our results demonstrated that the sheet web belongs to multiple origins, in contrast to those of previous studies. The reason may be similar to that found in orb webs. In conclusion, these results suggested that the evolutionary history of the web is more complex and difficult to unravel than previously thought.

### 4.6 Selection of Pressure Analysis

Mitochondrial PCGs are essential for aerobic metabolism. Selection pressure on relevant genes may shape their adaptation to extreme environments (Nielsen 2005). For example, the occurrence of positive selection on the *ND4*, *CYTB*, and *ATP8* genes in bats has been thought to be a dramatic change that enabled bats to meet their energetic requirements (Shen et al. 2010). Positive selection on *ATP6*, *ND2*, and *ND4* genes in galliforms may be associated with high‐altitude adaptation (Zhou, Shen, and Irwin 2014). The *ATP8* gene, which has been subjected to strong purifying selection, may be the main driver shaping mitogenomes diversity in deep‐sea corals (Wei et al. 2024). Our selective analysis revealed a significant positive selection signal for the *ATP8* gene in the *D. jiaxiangi* clade. Meanwhile, the *ATP6* and *ND1* genes of *D. jiaxiangi* clade and the *ND3*, *ND4L*, and *ND5* genes of the *A. aquatica* clade are accelerated evolutionary genes. Positive selection was detected for *ND4* and *ATP6* in spider mites (*Tetranychus truncates* and *Tetranychus pueraricola*). This process is thought to be associated with different modes of climatic adaptation (Sun, Jin, and Hoffmann 2018). Given that the *ND* gene encodes a peptide that constitutes mitochondrial complex I, it is directly involved in hydrogen and electron transfer in the respiratory chain and generates ATP through oxidative phosphorylation, which in turn is involved in energy metabolism. The *ATP* gene encodes ATP synthase, which is a key enzyme in energy synthesis. Amino acid changes within *ATP* and *ND* genes, which encode proteins that affect the efficiency of ATP synthesis (Sun, Jin, and Hoffmann 2018). As in high‐altitude environments for galliforms, anoxic aquatic environments imply a correlation between PSGs and adaptation to energy metabolism in aquatic environments (Zhou, Shen, and Irwin 2014, Chang et al. 2020). The *ATP8* and *ND6* genes with the highest mean *K*_a_/*K*_s_ values indicated increase in amino acid changes. This result is consistent with the results of previous studies (Chang et al. 2020; Kumar et al. 2020; Lv et al. 2018), which suggested that high *K*_a_/*K*_s_ values indicate that the *ATP8* gene had been subjected to relaxed selective constraints and accumulated mutations and therefore had lost its function (Chang et al. 2020) The *COX1* and *ND1* genes had the lowest mean *K*_a_/*K*_s_ values and thus may have been subjected to strong evolutionary pressure (Chang et al. 2020). Therefore, *COX1* has been widely used as a barcode marker for reconstructing genetic diversity in spiders and other taxa. In the yn00 model, the *K*_a_/*K*_s_ values of all 13 PCGs were less than 1 (Figure 7a), suggesting that purifying selection dominated the evolution of the spider mitogenome. This finding is consistent with the situation in spider mites (Sun, Jin, and Hoffmann 2018) and insects (Chang et al. 2020).

### 4.7 Convergent Evolution of Aquatic Environments

The role of mitochondria as sources of energy for cells may be related to the foraging strategy of spiders. Spiders' disorientation strategies include webbing and hunting. Webbing spiders use their webs as traps to catch prey, whereas hunting spiders must find prey by moving quickly (Foelix 2011). Thus, hunting spiders may require more energy to find prey than webbing spiders. *A. aquatica* and *D. jiaxiangi* are typical wandering hunting spiders. They need to stay underwater, where oxygen is limited, for long periods (McQueen and McLay 1983; Spagna, Crews, and Gillespie 2010), and thus they must use oxygen efficiently for energy metabolism to meet the demands for energy expenditure. In summary, positive or accelerated selection on spider mitochondrial *ATP* and *ND* genes may have shaped the evolution of aquatic spider lineages. In addition, genes are positively selected and accelerated for evolution in aquatic spider lineages, *A. aquatica* was concentrated in *ATP* genes, whereas *D. jiaxiangi* was concentrated in *ND* genes. This difference can be the result of different strategies evolved by the two aquatic spiders to adapt to freshwater and seawater environments. However, the *ND5* gene was detected by the JTT‐gene model and PCOC software as a convergent evolutionary gene in both aquatic spiders and may play an important role in adaptation to aquatic environments. Although our study did not directly test adaptation to aquatic environments, our results can inspire further studies on aquatic adaptation during spider evolution. Comparative genomic studies of spider lineages in Arachnida, the second largest group of arthropod taxa, remain largely underutilized because of limited data. Therefore, further increases in sample size, especially in aquatic spiders, are needed to reveal more information about molecular mechanisms underlying the adaptation of spiders to different environmental extremes.

## 5 Conclusions

Spiders play an important role in maintaining ecosystems, serving as the key members of the food chain, controlling pest populations by feeding on insects and other small organisms, and protecting crops and vegetation from pests and diseases. In addition, their position in the food web makes them a source of food for other animals, including birds, snakes, and mammals, which in turn maintain the balance of the ecosystem. In conclusion, the presence and activities of spiders in ecosystems are essential for maintaining biodiversity and ecological balance. Therefore, the origin and evolutionary pathways of spiders should be explored. In this study, we performed a systematic mitogenome analysis on the largest mitochondrial dataset of spiders to date, which comprises 255 species in 66 families. Our analysis enhances understanding of the taxonomic status, species diversity, distribution, mitochondrial characteristics, and genetic plasticity of spiders. Notably, our results suggested that spiders may have originated in the Devonian Nearctic realm and subsequently spread to the Palaearctic and Oriental realms and the orb web may have evolved from the original trodpar. Meanwhile, the extreme environmental factors in which spiders survive may have driven their evolution by inducing mutations in genes related to energy metabolism. However, this role needs to be further verified by collecting more environmental data.

## Author Contributions

**Rongxiang Zhang:** formal analysis (lead). **Niyan Xiang:** formal analysis (equal). **Xiaoman Gao:** data curation (lead). **Guiyu Zhang:** data curation (equal). **Tian Lu:** funding acquisition (lead), writing – review and editing (equal). **Tao Yuan:** formal analysis (lead), writing – original draft (lead), writing – review and editing (lead).

## Conflicts of Interest

The authors declare no conflicts of interest.

## Supporting information


**Figure S1.** The RSCU values for 76 spider mitochondrial protein‐coding genes.

**Figure S2.** ENc plotted against GC3s based on 13 PCGs.

**Figure S3.** (a) Phylogenetic relationships of 28 spider species. (b) ANI plots of 28 spider’s mitogenomes.

**Figure S4.** Estimating ancestral ranges of Zingiberaceae species using BioGeoBEARS and DEC+j model.

**Figure S5.** Ancestral trait reconstruction of web type in spiders using PastML.

**Figure S6.** Changes in the evolutionary rate of the *ATP6* gene during the evolution of 28 spider species.

**Appendix S1.** NCBI accession numbers for the 255 spider species and four outgroups used in this study.

**Appendix S2.** The 76 accession numbers for mitochondrial genome characterization in spiders.

**Appendix S3.** Alternative models and optimal partitioning strategies for 12 PCGs estimated using the Bayesian Information Criterion (BIC) in PartitionFinder.

**Appendix S4.** Dispersal rates of spiders in different geographic areas.

**Appendix S5.** Trait coding in different spider species.

**Appendix S6.** The 28 spiders were used to study mitochondrial evolutionary rates and selection pressure analysis.

**Appendix S7.** Mean mitochondrial genome length of 76 species of spiders from 27 families.

**Appendix S8.** The use of codons of 13 PCGs in 76 spiders.

**Appendix S9.** Summary of GC3s and ENC results for the mitochondrial genomes of 76 spider species.

**Appendix S10.** Statistics of RSCU values for PCGs in mitogenome from 6 families and all spiders.

**Appendix S11.** Gene arrangements in the mitogenomes of the spider.

**Appendix S12.** Summary of ANI analysis results for 28 spider’s mitogenomes.

**Appendix S13.** The tested biogeographic models in this study.

**Appendix S14.** Summary information on RASP results.

**Appendix S15.** Summary information on pastML results.

**Appendix S16.** *K*_a_/*K*_s_ for the 13 mitochondrial protein‐coding genes of the 28 spider mitogenomes examined.

**Appendix S17.** Convergent genes between the two aquatic spiders under the JTT‐Fgene model.

**Appendix S18.** Convergent genes were detected by the PCOC method between the two aquatic spiders.

## Data Availability

Sequence data that support the findings of this study can obtain in the National Center for Biotechnology Information (https://www.ncbi.nlm.nih.gov/sra/) via SRR6410545, SRR8519220, SRR8519225, SRR3932816, SRR15736564, SRR15736578, SRR16201431, SRR6410546, SRR16201447, SRR11292793, SRR13580908, SRR7363160, SRR16201442, SRR16201445, SRR11292747, SRR6410536, SRR6410537, SRR7363161, SRR11292791, SRR11292782, SRR11292767, SRR16201473, SRR16201440, SRR16201439, SRR11292754, SRR11292789, SRR6410532, SRR6410533, SRR11292772, SRR11292773, SRR16201470, SRR16201471, SRR7186688, SRR16201404, SRR11292751, and SRR11292750. The assembly mitogenomes presented in the study are available on figshare database (https://doi.org/10.6084/m9.figshare.27783108.v1).
