# Supplementary figures and images for: Molecular Phylogenetic Relationships Based on Mitogenomes of Spider: Insights Into Evolution and Adaptation to Extreme Environments
Source: Ecol Evol. 2025 Jan 7;15(1):e70774. doi: 10.1002/ece3.70774 (PMC11707259; doi:10.1002/ece3.70774)

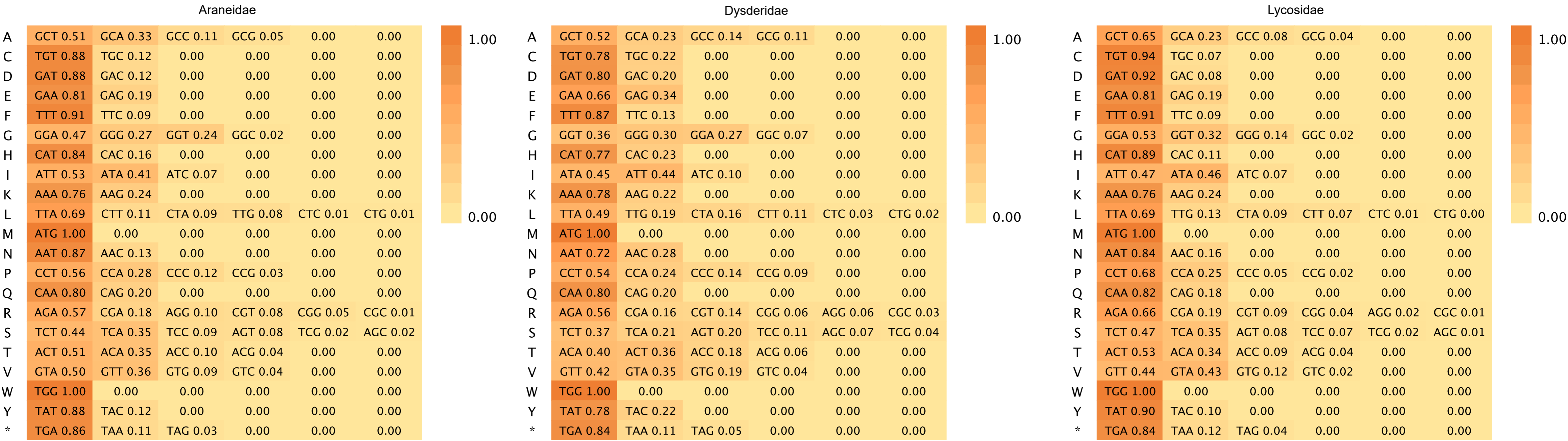

Lycosidae

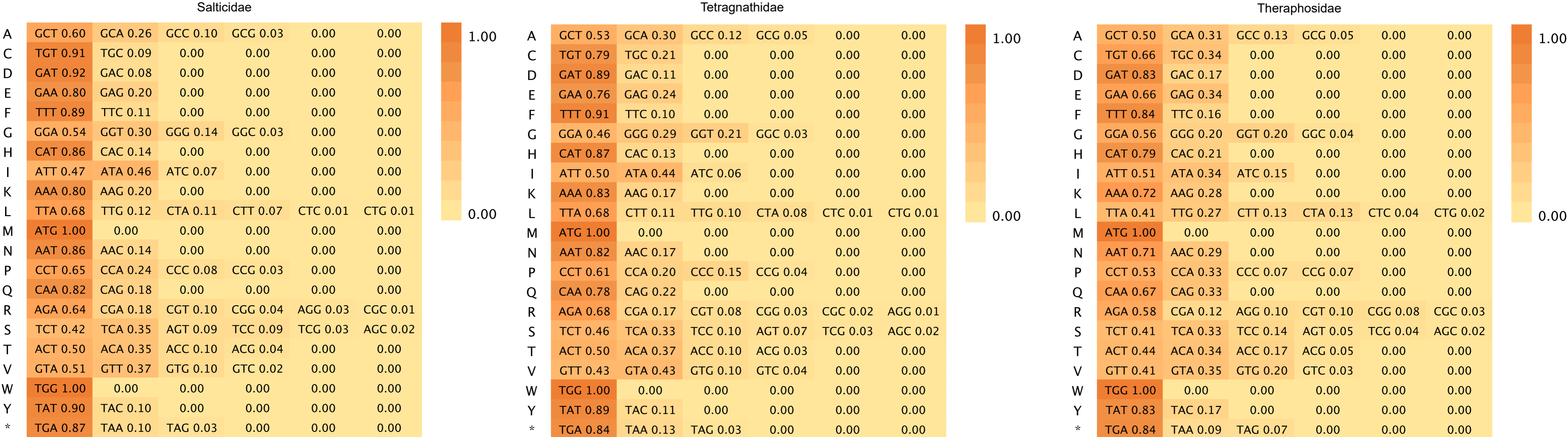

Theraphosidae

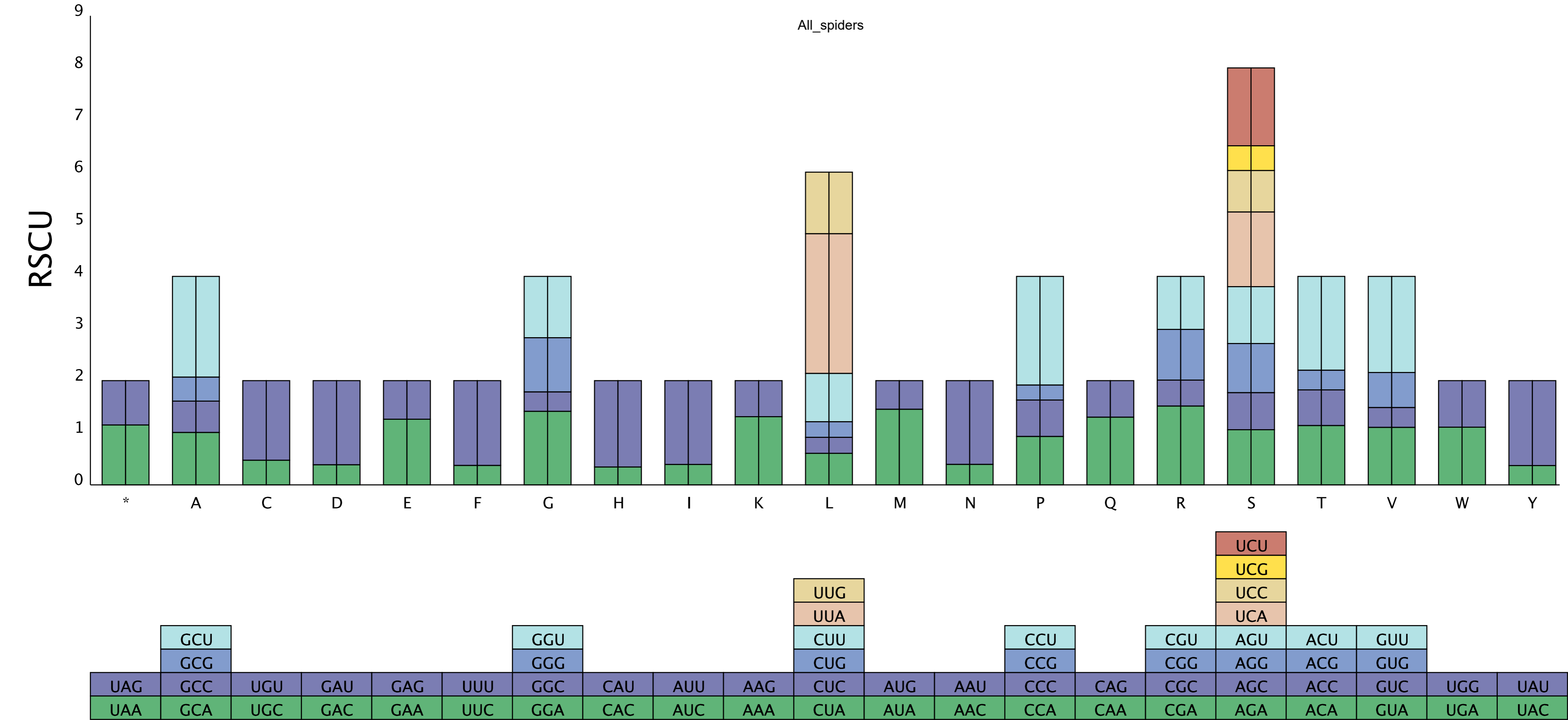

Supplement: Supplementary file 1 — Figure S1. The RSCU values for 76 spider mitochondrial protein‐coding genes. [file ECE3-15-e70774-s024.pdf]

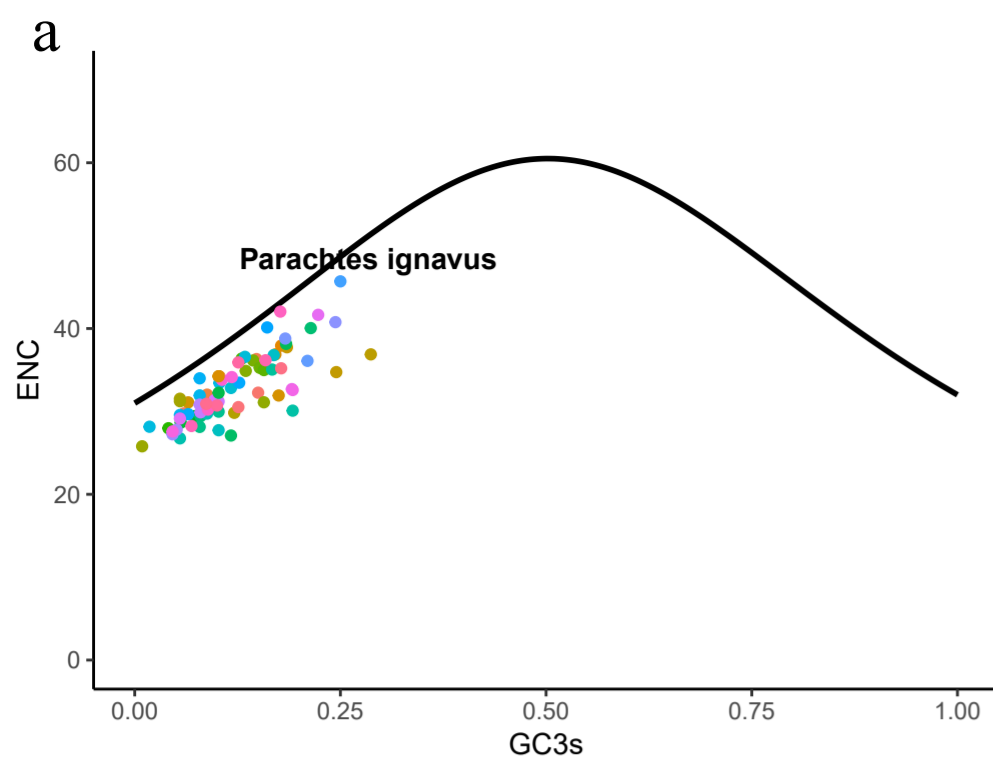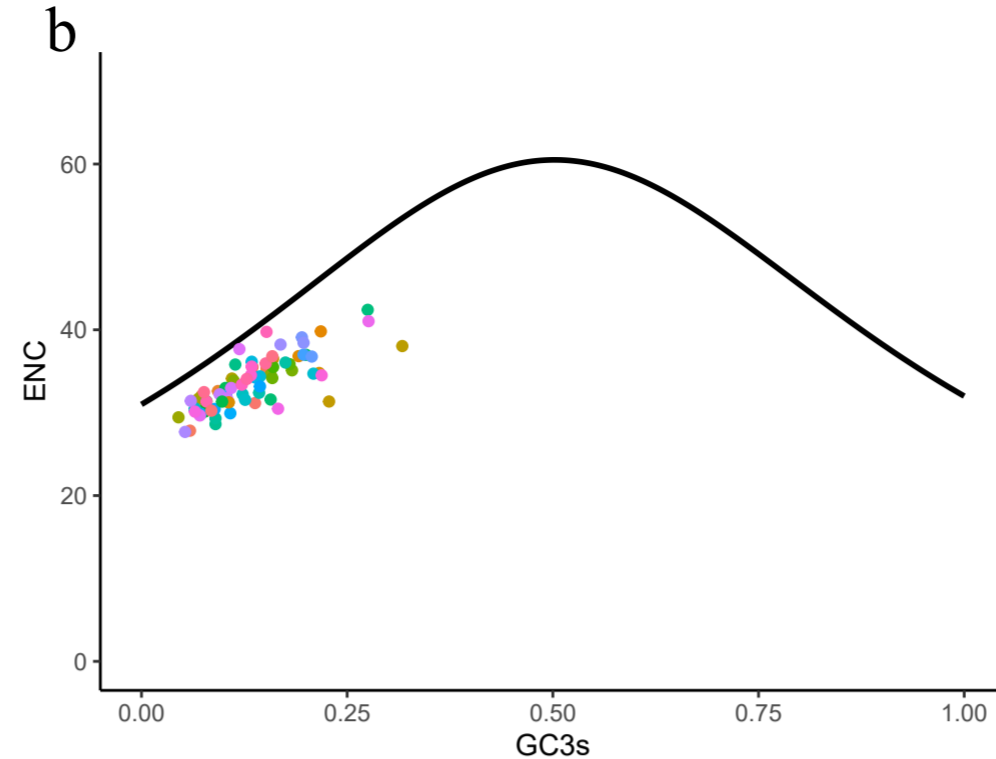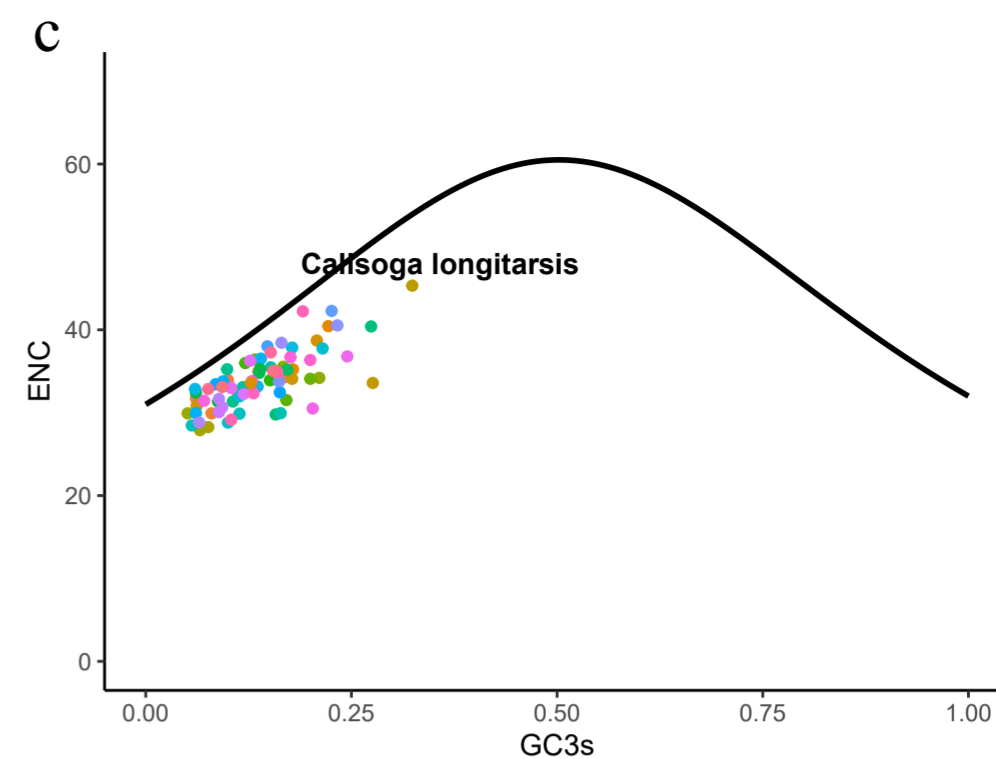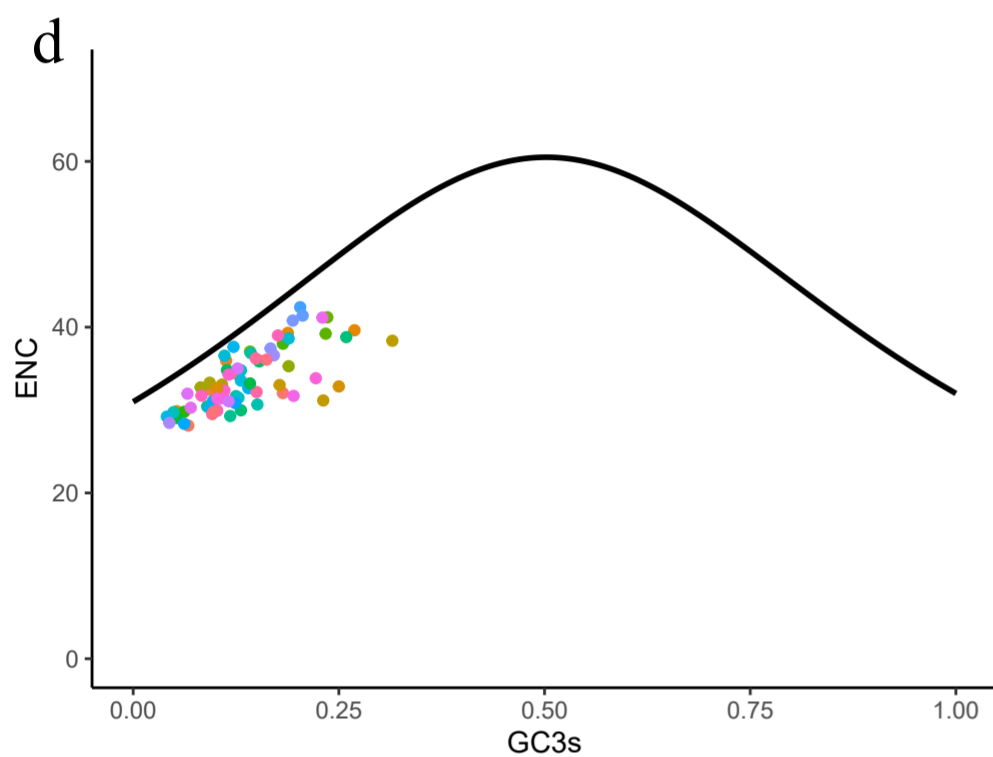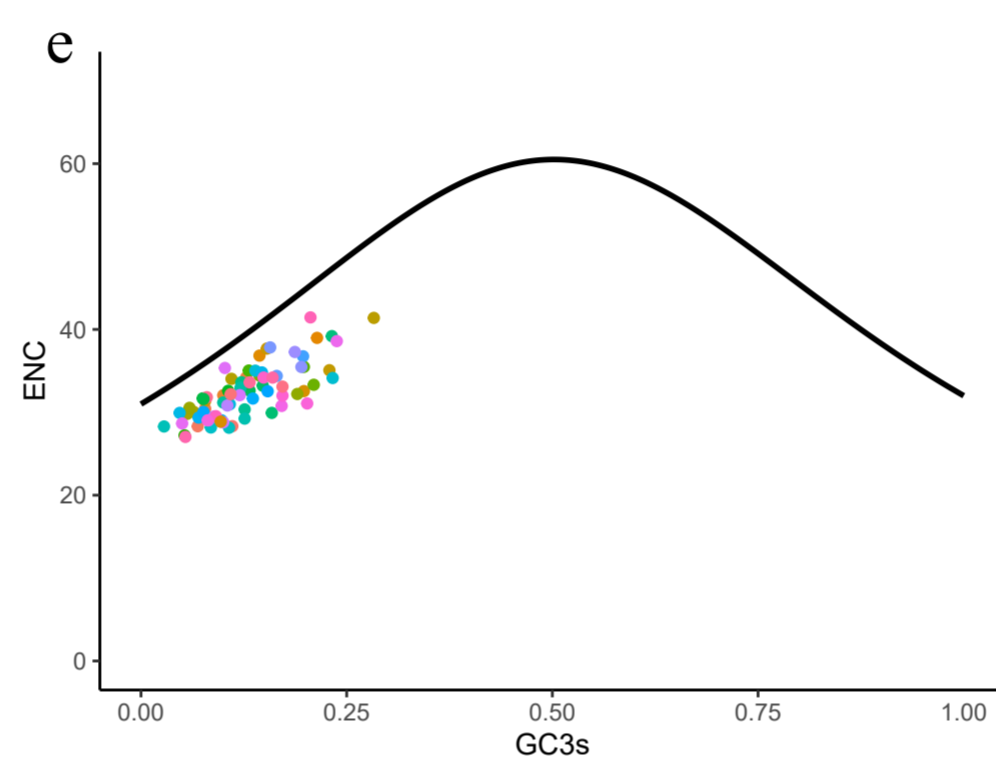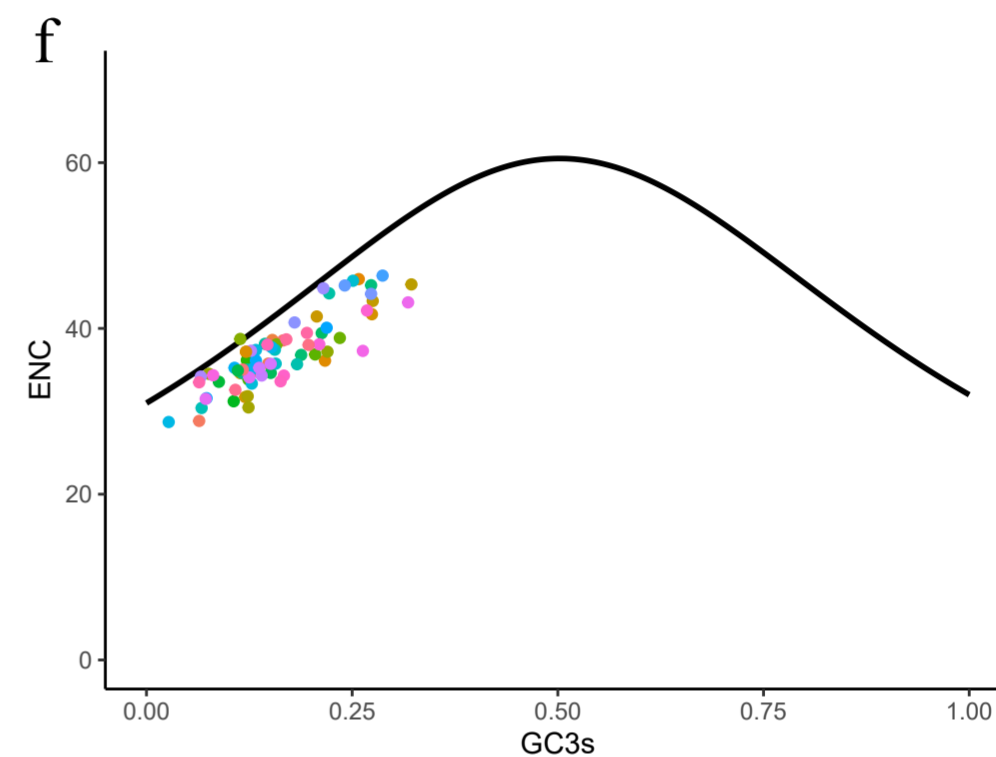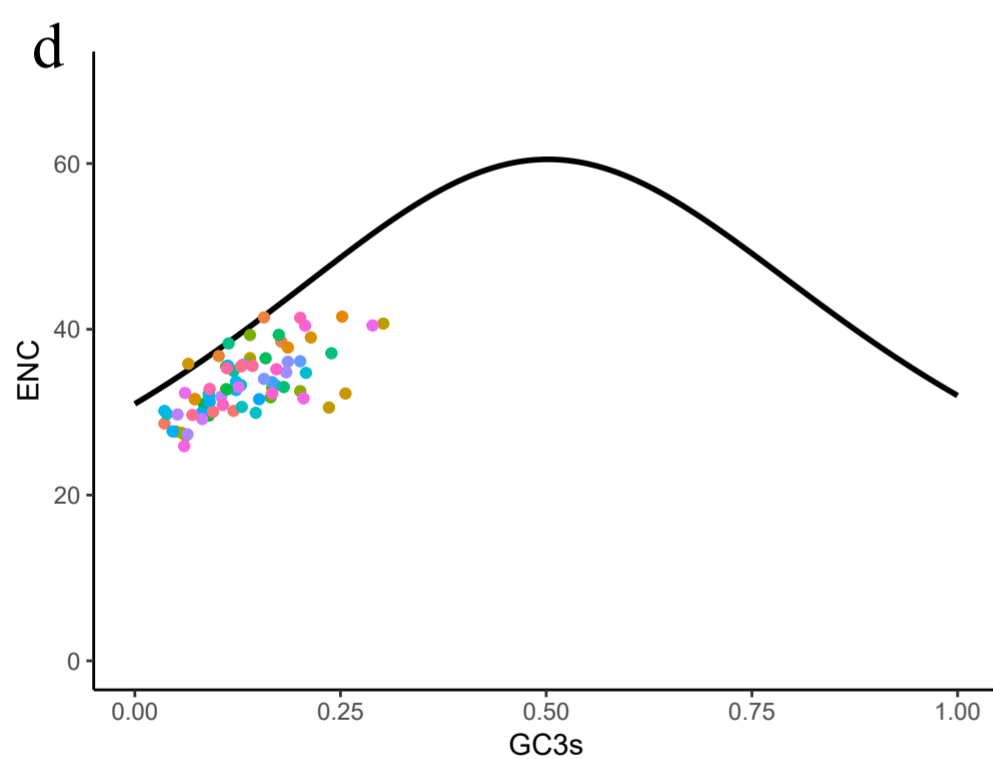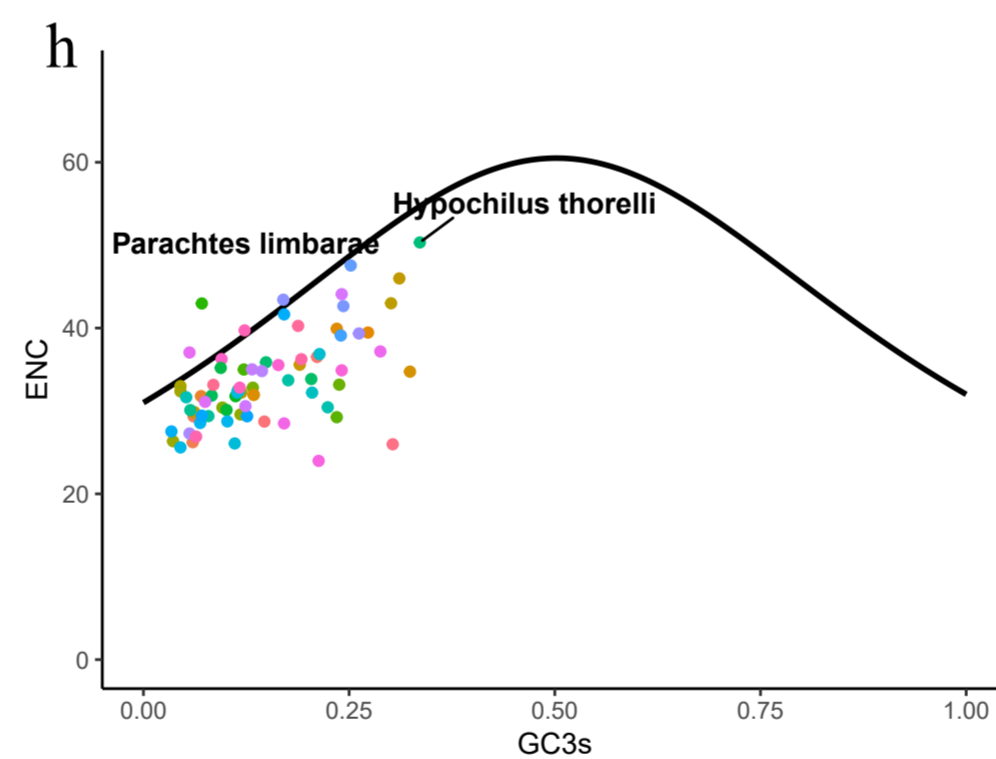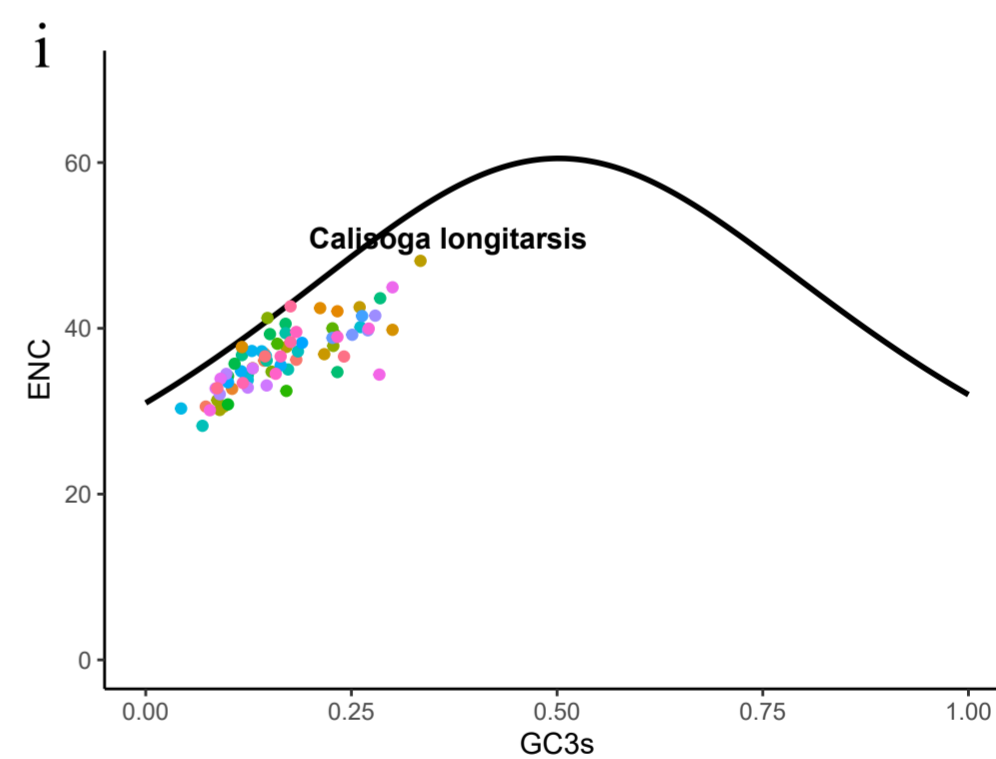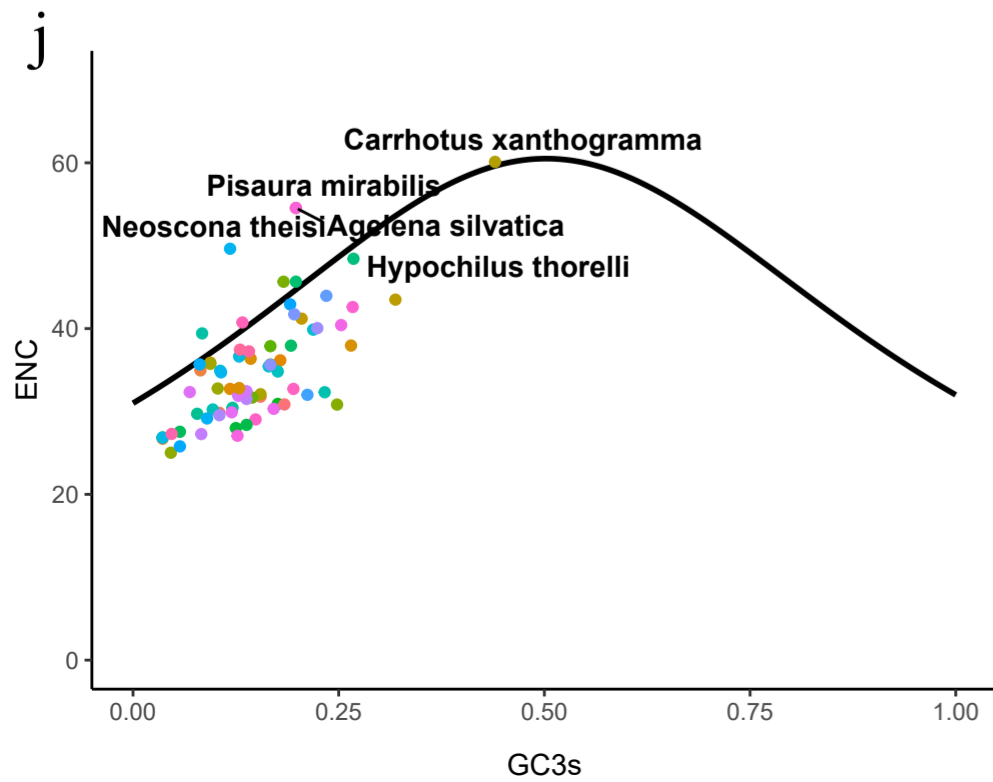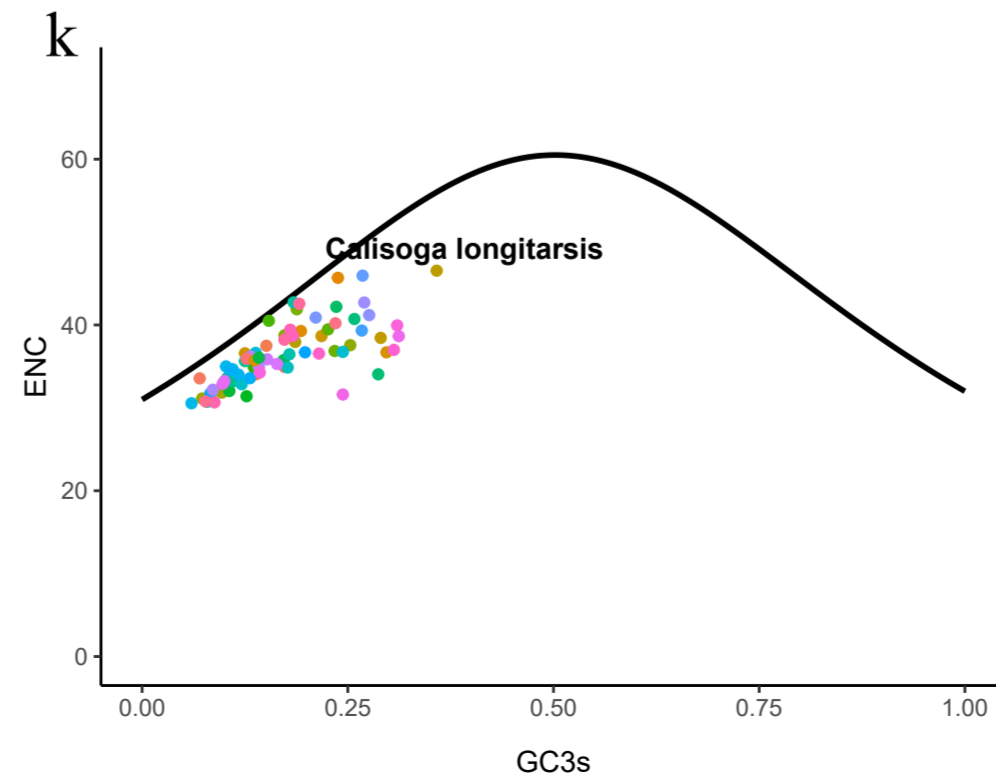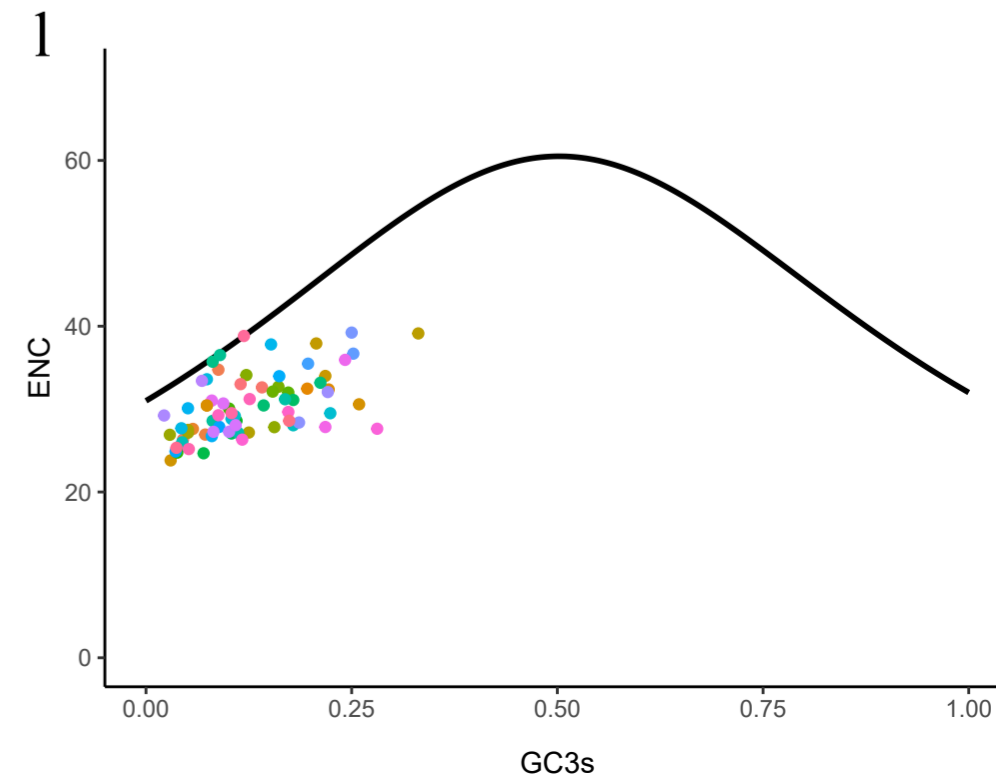

Supplement: Supplementary file 2 — Figure S2. ENc plotted against GC3s based on 13 PCGs. [file ECE3-15-e70774-s002.pdf]

(a)

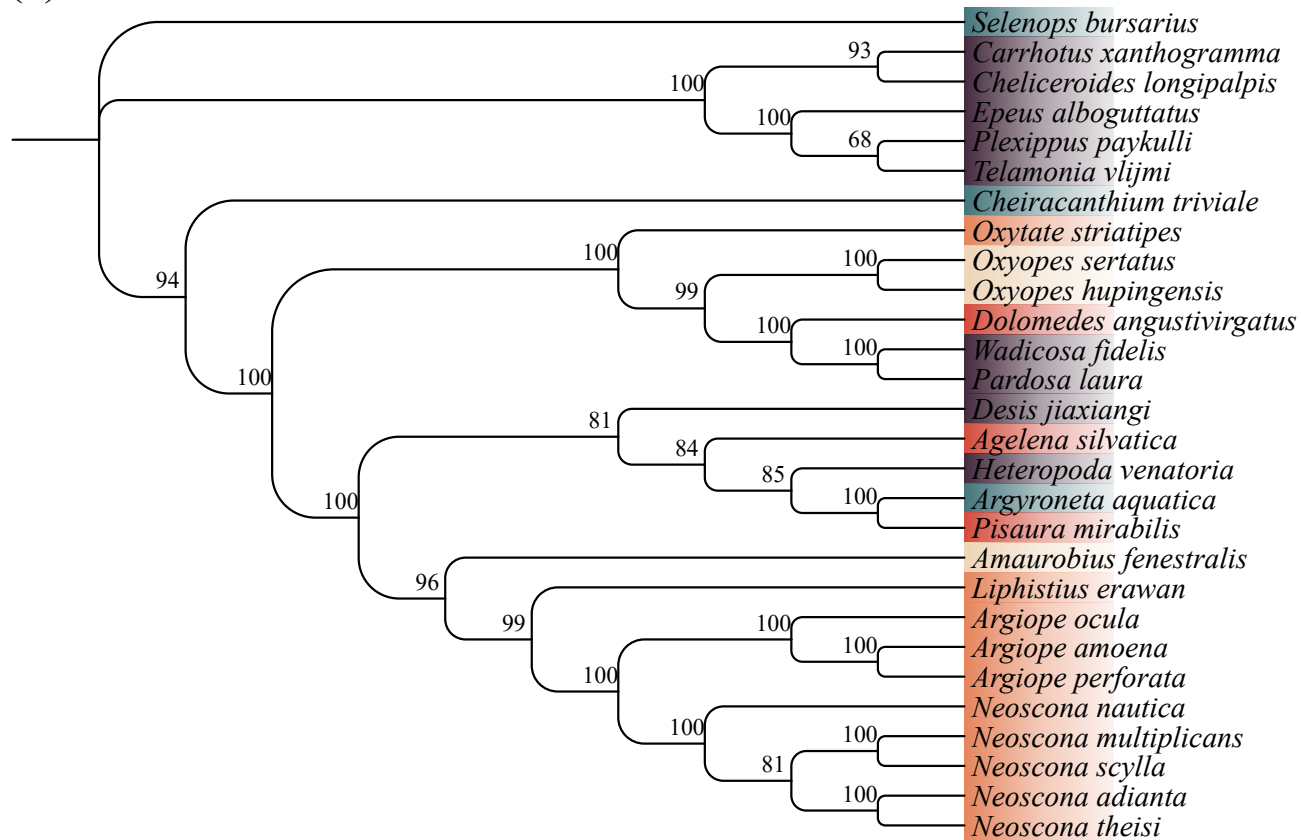

(b)

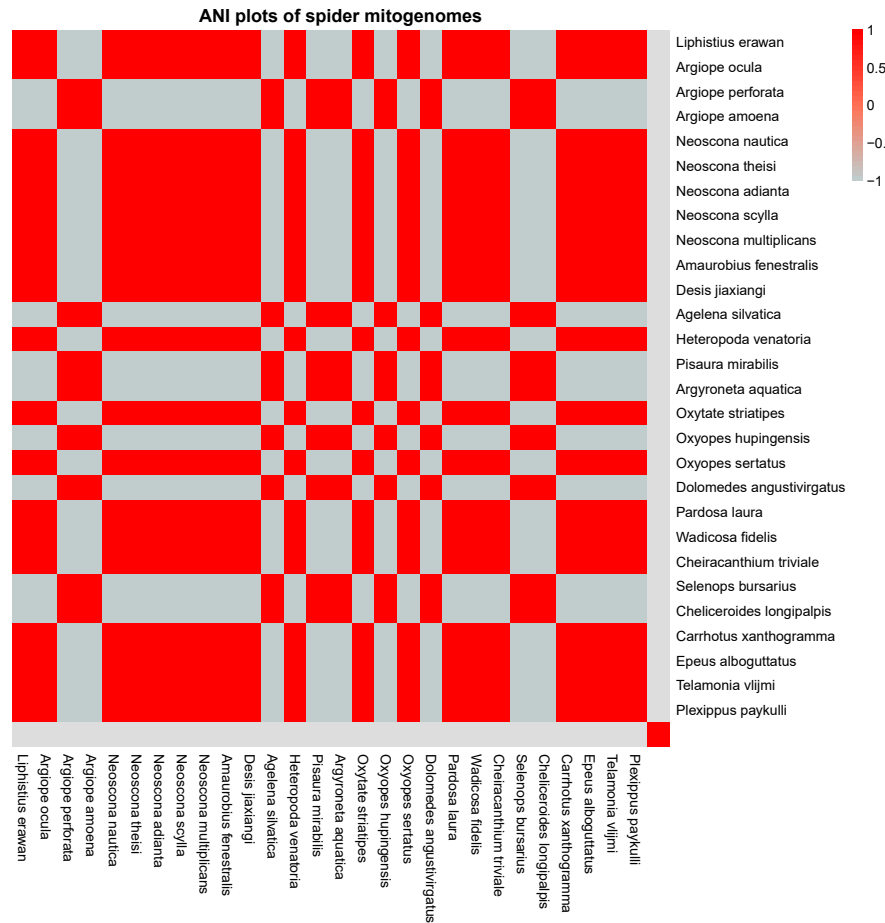

Supplement: Supplementary file 3 — Figure S3. (a) Phylogenetic relationships of 28 spider species. (b) ANI plots of 28 spider’s mitogenomes. [file ECE3-15-e70774-s016.pdf]

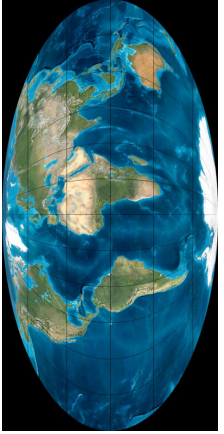

now

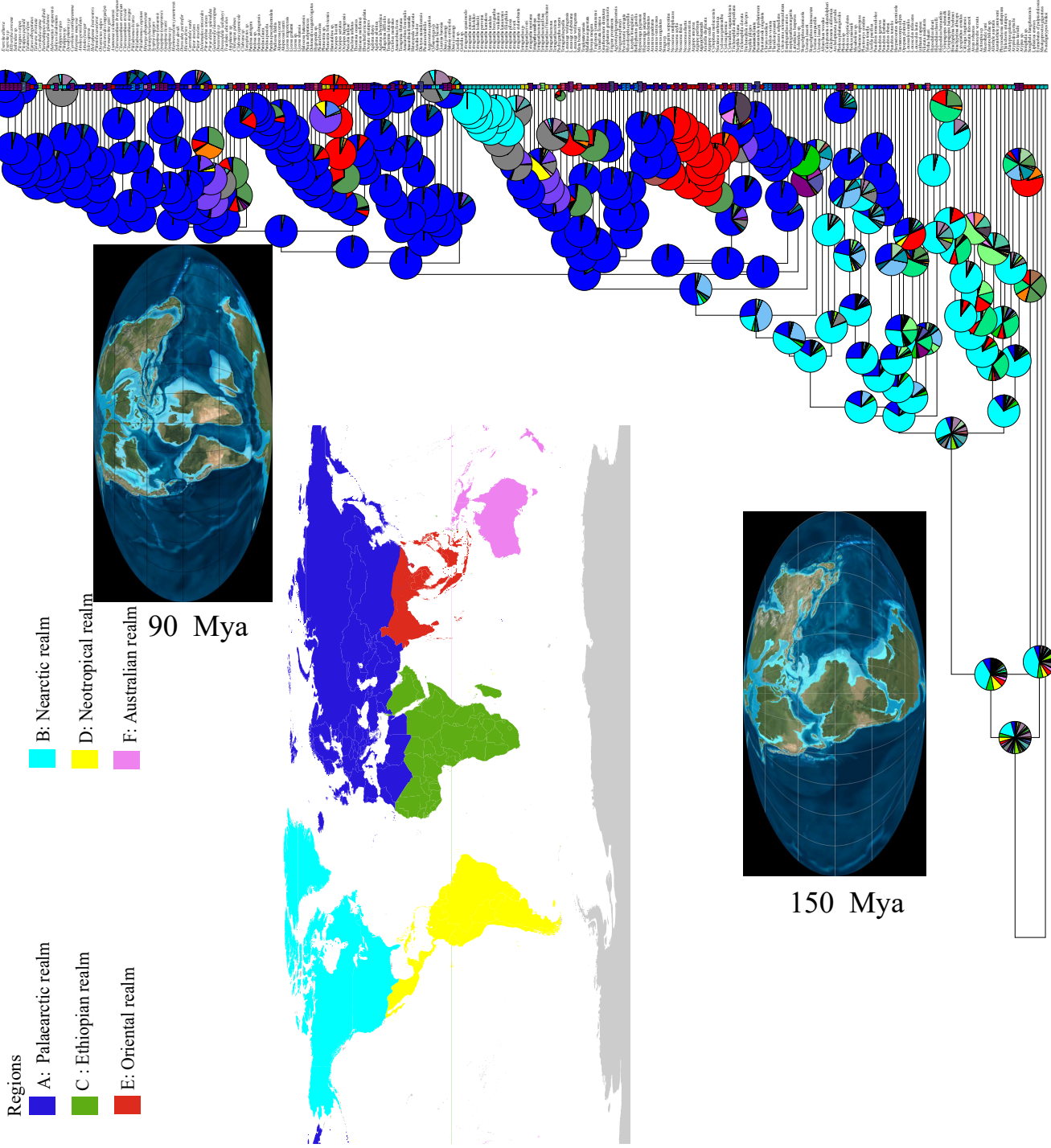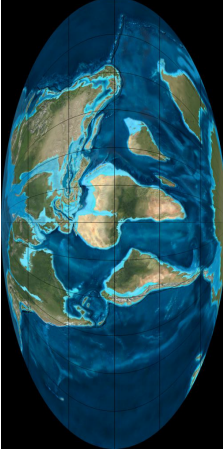

65 Mya

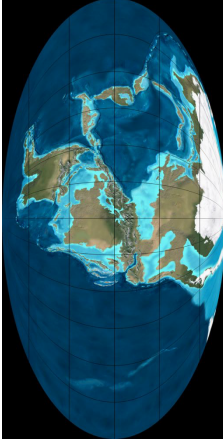

400 Mya

Supplement: Supplementary file 4 — Figure S4. Estimating ancestral ranges of Zingiberaceae species using BioGeoBEARS and DEC+j model. [file ECE3-15-e70774-s009.pdf]

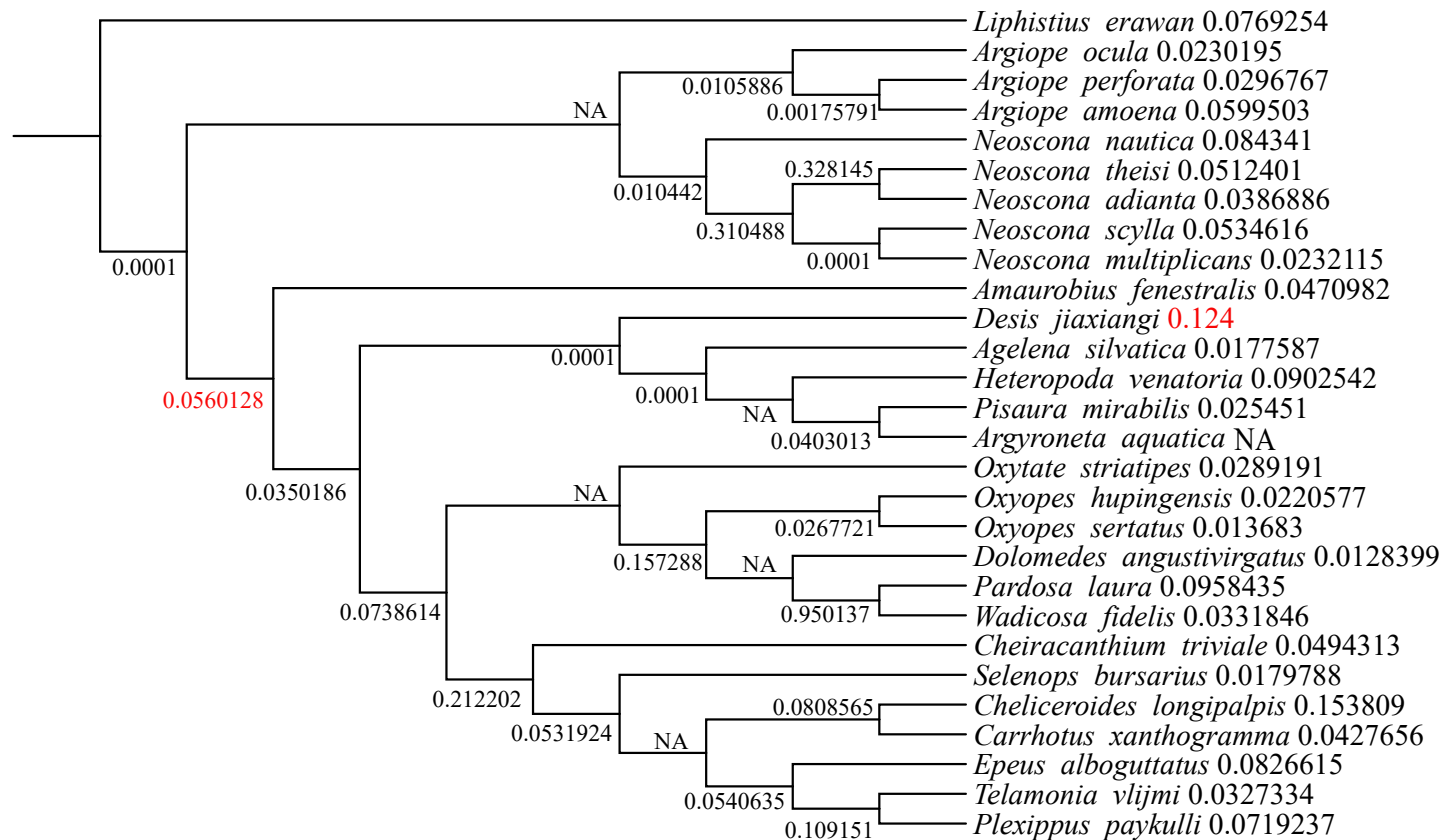

Supplement: Supplementary file 6 — Figure S6. Changes in the evolutionary rate of the ATP6 gene during the evolution of 28 spider species. [file ECE3-15-e70774-s019.pdf]
