# Supplementary material for: Molecular Phylogenetic Relationships Based on Mitogenomes of Spider: Insights Into Evolution and Adaptation to Extreme Environments
Source: Ecol Evol. 2025 Jan 7;15(1):e70774. doi: 10.1002/ece3.70774 (PMC11707259; doi:10.1002/ece3.70774)
Supplement: Supplementary file 5 — Figure S5. Ancestral trait reconstruction of web type in spiders using PastML. [file ECE3-15-e70774-s022.pdf]

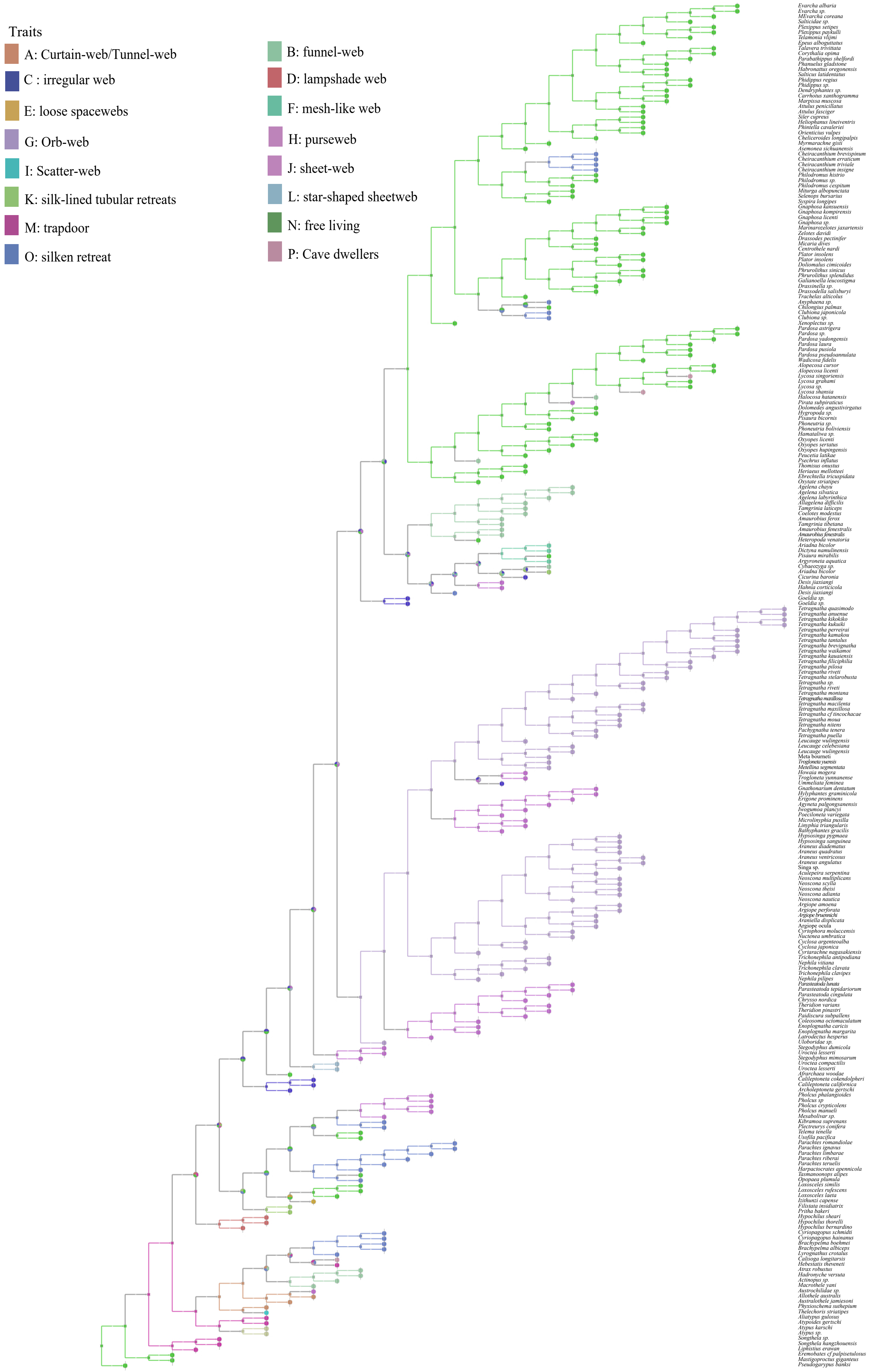

Traits
A: Curtain-web/Tunnel-web
C : irregular web
E: loose spacewebs
G: Orb-web
I: Scatter-web
K: silk-lined tubular retreats
M: trapdoor
O: silken retreat
B: funnel-web
D: lampshade web
F: mesh-like web
H: purseweb
J: sheet-web
L: star-shaped sheetweb
N: free living
P: Cave dwellers
Evarcha albaria
Evarcha sp.
MEvarcha coreana
Salticidae sp.
Plexippus setipes
Plexippus paykulli
Telamonia vlijmi
Epeus alboguttatus
Talavera trivittata
Corythalia opima
Parabathippus shelfordi
Phanuelus gladstone
Habronattus oregonensis
Salticus latidentatus
Phidippus regius
Phidippus sp.
Dendryphantes sp.
Carrhotus xanthogramma
Marpissa muscosa
Attulus penicillatus
Attulus fasciger
Siler cupreus
Heliophanus lineiventris
Phintella cavaleriei
Orienticius vulpes
Cheliceroides longipalpis
Myrmarachne gisti
Asemonea sichuanensis
Cheiracanthium brevispinum
Cheiracanthium erraticum
Cheiracanthium triviale
Cheiracanthium insigne
Philodromus histrio
Philodromus sp.
Philodromus cespitum
Miturga albopunctata
Selenops bursarius
Syspira longipes
Gnaphosa kansuensis
Gnaphosa kompirensis
Gnaphosa licenti
Gnaphosa sp.
Marinarozelotes jaxartensis
Zelotes davidi
Drassodes pectinifer
Micaria dives
Centrothele nardi
Plator insolens
Plator insolens
Doliomalus cimicoides
Phrurolithus sinicus
Phrurolithus splendidus
Galianoella leucostigma
Drassinella sp.
Drassodella salisburyi
Trachelas alticolus
Anyphaena sp.
Chilongius palmas
Clubiona japonicola
Clubiona sp.
Xenoplectus sp.
Pardosa astrigera
Pardosa sp.
Pardosa yadongensis
Pardosa laura
Pardosa pusiola
Pardosa pseudoannulata
Wadicosa fidelis
Alopecosa cursor
Alopecosa licenti
Lycosa singoriensis
Lycosa grahami
Lycosa sp.
Lycosa shansia
Halocosa hatanensis
Pirata subpiraticus
Dolomedes angustivirgatus
Hygropoda sp.
Pisaura bicornis
Phoneutria sp.
Phoneutria boliviensis
Hamataliwa sp.
Oxyopes licenti
Oxyopes sertatus
Oxyopes hupingensis
Peucetia latikae
Psechrus inflatus
Thomisus onustus
Heriaeus mellotteei
Ebrechtella tricuspidata
Oxytate striatipes
Agelena chayu
Agelena silvatica
Agelena labyrinthica
Allagelena difficilis
Tamgrinia laticeps
Coelotes modestus
Amaurobius ferox
Tamgrinia tibetana
Amaurobius fenestralis
Amaurobius fenestralis
Heteropoda venatoria
Ariadna bicolor
Dictyna namulinensis
Pisaura mirabilis
Argyroneta aquatica
Cybaeozyga sp.
Ariadna bicolor
Cicurina baronia
Desis jiaxiangi
Hahnia corticicola
Desis jiaxiangi
Goeldia sp.
Goeldia sp.
Tetragnatha quasimodo
Tetragnatha anuenue
Tetragnatha kikokiko
Tetragnatha kukuiki
Tetragnatha perreirai
Tetragnatha kamakou
Tetragnatha tantalus
Tetragnatha brevignatha
Tetragnatha waikamoi
Tetragnatha kauaiensis
Tetragnatha filiciphilia
Tetragnatha pilosa
Tetragnatha riveti
Tetragnatha stelarobusta
Tetragnatha sp.
Tetragnatha riveti
Tetragnatha montana
Tetragnatha maxillosa
Tetragnatha macilenta
Tetragnatha maxillosa
Tetragnatha cf tincochacae
Tetragnatha moua
Tetragnatha nitens
Pachygnatha tenera
Tetragnatha puella
Leucauge wulingensis
Leucauge celebesiana
Leucauge wulingensis
Meta bourneti
Trogloneta yuensis
Metellina segmentata
Howaia mogera
Trogloneta yunnanense
Ummeliata feminea
Gnathonarium dentatum
Hylyphantes graminicola
Erigone prominens
Agyneta palgongsanensis
Iwogumoa plancyi
Poeciloneta variegata
Microlinyphia pusilla
Linyphia triangularis
Bathyphantes gracilis
Hypsosinga pygmaea
Hypsosinga sanguinea
Araneus diadematus
Araneus quadratus
Araneus ventricosus
Araneus angulatus
Singa sp.
Aculepeira serpentina
Neoscona multiplicans
Neoscona scylla
Neoscona theisi
Neoscona adianta
Neoscona nautica
Argiope amoena
Argiope perforata
Argiope bruennichi
Araniella displicata
Argiope ocula
Cyrtophora moluccensis
Nuctenea umbratica
Cyclosa argenteoalba
Cyclosa japonica
Cyrtarachne nagasakiensis
Trichonephila antipodiana
Nephila vitiana
Trichonephila clavata
Trichonephila clavipes
Nephila pilipes
Parasteatoda lunata
Parasteatoda tepidariorum
Parasteatoda cingulata
Chrysso nordica
Theridion varians
Theridion pinastri
Paidiscura subpallens
Coleosoma octomaculatum
Enoplognatha caricis
Enoplognatha margarita
Latrodectus hesperus
Uloboridae sp.
Stegodyphus dumicola
Uroctea lesserti
Stegodyphus mimosarum
Uroctea compactilis
Uroctea lesserti
Afrarchaea woodae
Calileptoneta cokendolpheri
Calileptoneta californica
Archoleptoneta gertschi
Pholcus phalangioides
Pholcus sp
Pholcus crypticolens
Pholcus manueli
Mesabolivar sp.
Kibramoa suprenans
Plectreurys conifera
Telema tenella
Usofila pacifica
Parachtes romandiolae
Parachtes ignavus
Parachtes limbarae
Parachtes riberai
Parachtes teruelis
Harpactocrates apennicola
Tasmanoonops alipes
Opopaea plumula
Loxosceles similis
Loxosceles rufescens
Loxosceles laeta
Izithunzi capense
Filistata insidiatrix
Pritha bakeri
Hypochilus sheari
Hypochilus thorelli
Hypochilus bernardino
Cyriopagopus schmidti
Cyriopagopus hainanus
Brachypelma boehmei
Brachypelma albiceps
Lyrognathus crotalus
Calisoga longitarsis
Hebestatis theveneti
Atrax robustus
Hadronyche versuta
Actinopus sp.
Macrothele yani
Austrochilidae sp.
Allothele australis
Australothele jamiesoni
Phyxioschema suthepium
Thelechoris striatipes
Aliatypus gulosus
Atypoides gertschi
Atypus karschi
Atypus sp.
Songthela sp.
Songthela hangzhouensis
Liphistius erawan
Eremobates cf palpisetulosus
Mastigoproctus giganteus
Pseudogarypus banksi
